# Gene network inference using continuous time Bayesian networks: a comparative study and application to Th17 cell differentiation

**DOI:** 10.1186/s12859-014-0387-x

**Published:** 2014-12-11

**Authors:** Enzo Acerbi, Teresa Zelante, Vipin Narang, Fabio Stella

**Affiliations:** Singapore Immunology Network (SIgN), A*STAR, 8A Biomedical Grove, Immunos Building, Level 4, 138648 Singapore; School of Translational and Molecular Medicine (DIMET), University of Milan-Bicocca, Milan, Italy; Department of Informatics, Systems and Communication, University of Milano-Bicocca, Viale Sarca 336, Building U14, Milan, 20126 Italy

**Keywords:** Gene network reconstruction, Time course, Continuous time Bayesian network

## Abstract

**Background:**

Dynamic aspects of gene regulatory networks are typically investigated by measuring system variables at multiple time points. Current state-of-the-art computational approaches for reconstructing gene networks directly build on such data, making a strong assumption that the system evolves in a synchronous fashion at fixed points in time. However, nowadays omics data are being generated with increasing time course granularity. Thus, modellers now have the possibility to represent the system as evolving in continuous time and to improve the models’ expressiveness.

**Results:**

Continuous time Bayesian networks are proposed as a new approach for gene network reconstruction from time course expression data. Their performance was compared to two state-of-the-art methods: dynamic Bayesian networks and Granger causality analysis. On simulated data, the methods comparison was carried out for networks of increasing size, for measurements taken at different time granularity densities and for measurements unevenly spaced over time. Continuous time Bayesian networks outperformed the other methods in terms of the accuracy of regulatory interactions learnt from data for all network sizes. Furthermore, their performance degraded smoothly as the size of the network increased. Continuous time Bayesian networks were significantly better than dynamic Bayesian networks for all time granularities tested and better than Granger causality for dense time series. Both continuous time Bayesian networks and Granger causality performed robustly for unevenly spaced time series, with no significant loss of performance compared to the evenly spaced case, while the same did not hold true for dynamic Bayesian networks. The comparison included the IRMA experimental datasets which confirmed the effectiveness of the proposed method. Continuous time Bayesian networks were then applied to elucidate the regulatory mechanisms controlling murine T helper 17 (Th17) cell differentiation and were found to be effective in discovering well-known regulatory mechanisms, as well as new plausible biological insights.

**Conclusions:**

Continuous time Bayesian networks were effective on networks of both small and large size and were particularly feasible when the measurements were not evenly distributed over time. Reconstruction of the murine Th17 cell differentiation network using continuous time Bayesian networks revealed several autocrine loops, suggesting that Th17 cells may be auto regulating their own differentiation process.

## Background

In response to internal and external stimuli, a cell modifies its transcriptional state through the activation of multiple regulatory interactions that take place over time and which include complex mechanisms such as regulation chains, auto-regulations and feedback loops. Understanding gene regulatory networks (GRNs) is of extreme relevance in molecular biology and represents an open challenge for computational sciences. The task of uncovering the underlying causal structure of these cellular dynamics is referred to as gene network reconstruction or *(network)* “*reverse-engineering*”.

Reconstruction of gene regulatory networks from time course expression data is an active area of research [1,2]. In recent years, the granularity and length of time course data made available by omics technologies has been constantly increasing. This offers a chance for a deep study of the dynamic evolution of regulatory networks [3] and calls for computational approaches that can effectively exploit the dynamic nature of data. In fact, most of the state-of-the-art methodologies for gene network reconstruction have been conceived before the advent of omic technologies and may not be always suitable for the new types and magnitudes of data.

A number of approaches have been applied to the GRNs reconstruction problem. Boolean networks [4] have been widely applied but are now giving way to more sophisticated approaches. Probabilistic graphical models such as Bayesian Networks [5] were shown to be powerful tools for solving the GRN reconstruction problem [6] and they led to significant discoveries [7]. When richer time course measurements started to be made available, Dynamic Bayesian networks (DBNs) [8] gained more and more relevance in the field, and today are largely applied with many variations and proposed optimizations. Other probabilistic approaches are state space models [9] and probabilistic Boolean networks [10]; however it has been shown that the latter are outperformed by DBNs for GRN reconstruction problems [11]. Other approaches are ordinary differential equations (ODEs) [12,13] which tend to become infeasible as the size of the network increases. Information-theoretic algorithms such as ARACNE [14] led to interesting discoveries [15], as well as evolutionary algorithms, which are reviewed in [16]. Finally, Granger causality (GC) [17,18] is a robust method for analysing time course data; since its early introduction it has been successfully applied to a multitude of domains such as economics, neuroscience and biology. Exhaustive reviews of the existing network reconstruction approaches can be found in [19-23].

Dynamic aspects of regulatory networks are investigated by measuring the system variables at multiple time points (e.g. through gene expression microarray or mRNA sequencing). This approach is the result of technological constraints of the experimental techniques which only allow for measurements of “snapshots” of the system at multiple time points. In this situation the risk of missing important pieces of information is high if the sample rate is not adequately chosen or not fine enough (issue known as temporal aggregation bias). While this issue is currently unavoidable, when computationally analyzing these time course datasets it can be advantageous to separate the way the time course data is experimentally obtained from the way the time is represented in the computational model. Current state-of-the-art approaches described above directly build on “snapshot-like” data, making the strong assumption that the system under investigation evolves in a synchronous fashion at fixed points in time. Even when only discrete time data is available, modeling the system as continuously evolving over time represents a conceptually more correct/natural approximation and improves model expressiveness [24]. Nowadays, the finely grained time course data made available by high throughput technologies make this continuous time representation feasible. It is also relevant to note that time course data are often unevenly spaced (measurements are not taken at equal width intervals). In such situations a continuous time model is preferable as it makes the analysis independent of the data sampling intervals.

In this paper continuous time Bayesian networks (CTBNs) [25] are proposed as a new approach for GRN reconstruction from time course data. In a CTBN variables can evolve continuously over time as a function of a continuous time conditional Markov process while the efficient factored state representation derives from the theory of Bayesian networks. Such setting brings many advantages to the description of the temporal aspect of a system, some of them directly relevant to the GRN reconstruction task. Firstly, the structural learning problem for CTBNs can be solved locally and in polynomial time with respect to the dimension of the dataset once the maximum number of regulators for each gene is set. This feature suits regulatory networks well, which are systems characterized by a large number of variables (genes) and where genes are typically regulated only by a limited number of other genes [26]. The second advantage is that CTBNs can naturally handle variables evolving at different time granularities. Gene networks are characterized by the presence of both regulatory interactions which happen quickly, e.g. within minutes from a given triggering event, as well as interactions which take place at a slower pace, e.g. within hours or days. To reconstruct such regulatory networks, one may want to integrate data coming from experiments measuring genes whose state evolve at different rates. In such a context, CTBNs is naturally able to learn the overall causal network by combining data coming from different time granularities. The third advantage is that once the network structure and parameters have been inferred, through inference CTBNs can answer queries directly involving the quantification of the temporal aspects such as *“for how long does gene X have to remain up-regulated to have an effect on the regulation on gene Y?”* and in presence of partial evidence such as *“What is the most probable state for gene X at time t given that I observed that gene Y was up-regulated from time t - α to t - β ?”*. With their graphical representation of causal relations, CTBNs also provide an intuitive and meaningful level of abstraction of dynamic regulatory process which can help a molecular biologist to gain a better understanding of the systems studied. Finally, CTBNs conserve all of the advantages which are characteristic of probabilistic graphical models and which make them suitable for the analysis of biological networks [27].

The effectiveness of CTBNs for GRN reconstruction is verified through a comparison with two state-of-the-art models, namely DBNs and GC, in the case where no *a priori* knowledge about the system is available. Both DBNs and GC do not implement a direct representation of time. DBNs are built on the observational model assumption, with time slices representing the status of the system at evenly spaced time points. Hence, if data samples are not collected at fixed width intervals one must either choose a time granularity equal to the smallest time interval between measurements or bias the data by imposing a uniform time granularity: in the first case the computational cost may increase dramatically while the second solution can lead to biased results. Moreover, due to the presence of intra-slice arcs for which the acyclicity constraint must be respected, learning DBNs in their general formulation is a NP-hard problem. GC implements a type of analysis based on an autoregressive model aimed to test if knowledge about the past values of a variable can help in predicting the future value of another variable. GC has a great historical and current relevance when faced with the task of inferring causal relations from time series data. Its simplicity, flexibility and effectiveness made it broadly applied. However, almost all GC tests assume that the time intervals between measurements are fixed, risking to obtain biased results if this assumption is not verified. GC is designed to work on continuous valued variables, while DBNs have been developed to analyze continuous or discrete valued variables. A drawback of CTBNs is that they have been developed only to analyze discrete valued quantities. DBNs and GC were directly compared for the reconstruction of gene networks in [28]: the authors showed that when the length of the time course is smaller than a given threshold, DBNs tend to outperform GC while vice-versa when the length of the time course is greater than a threshold. CTBNs theoretically overcome the limitations associated with the discrete-time assumptions of both DBNs and GC. Therefore, we had reason to believe that CTBNs would show advantages over DBNs and GC when applied to the problem of gene network reconstruction.

The analysis and comparisons performed here are based on an extensive and robust set of numerical experiments run on simulated time course data and include a test on an experimental dataset as well. The study with simulated data has been conducted on networks of 10, 20, 50 and 100 genes in order to investigate how the approaches perform on systems of increasing size; the networks were extracted from the known transcriptional networks of two different organisms: *E. coli* and *S. cerevisiae*. To ensure robustness the performance is not calculated on a single network instance, but it is estimated by the average value computed over a set of 10 randomly sampled network instances of the same size.

We then investigated the methods’ performances with respect to different time course granularities (11, 21 and 31 time points), while keeping the overall time duration of the experiment fixed. Finally, we investigated how the methods perform when the measurements are collected at unevenly spaced time points. For a robust comparison we evaluated the performance on 10 different random time point instances. Our comparative investigation also included an experimental dataset as well: a 5 genes regulatory network synthetically constructed in the yeast *S. cerevisiae* (IRMA network) [29] which provided rich time course expression data and a gold standard for accurate benchmarking. In the second part of this work, we applied CTBNs for the reconstruction of the regulatory network responsible for murine T helper 17 (Th17) cell differentiation, testing their ability to confirm known regulatory interactions and to generate new plausible biological insights.

## Methods

### Dynamic Bayesian networks

The definition of DBN has necessarily to start from the definition of a Bayesian network. A Bayesian network (BN) is a graphical model consisting of two components - a causal graph (qualitative component) which encodes conditional dependence and independence relationships between the variables (nodes), and a set of conditional probability tables (CPTs) (quantitative component) quantifying how strong the influence is of one variable over the others. More formally:

#### **Definition1**.

(Bayesian Network). [30] A BN consists of:

- A set of random variables (nodes) and a set of oriented arcs connecting the random variables which form a Direct Acyclic Graph (DAG).
- A finite set of mutually exclusive states associated with each random variable.
- For each random variable *X* with parents *Y*_1_,…,*Y*_*n*_ a CPT encoding the probability *P*=(*X*|*Y*_1_,…,*Y*_*n*_). In other words, the CPT quantifies the effect of the parents *Y*_1_,…,*Y*_*n*_ on *X*. If *X* has no parents, *X* is associated with an unconditional probability table, that is *P*(*X*).

Exploiting the concept of conditional independence, a BN compactly represents the joint probability distribution over a set of random variables by factorizing it into a product of conditional distributions contained in the CPTs associated with each node in the graph.

Learning a BN involves:

- Parameter learning: learning of the conditional probability distributions.
- Structural learning: learning of the qualitative component of the network, e.g. the relations of conditional independence between variables.
- The goal of the learning phase is the finding of the structure and the parameters which best describe the initial data.

Bayesian networks are a static model, since variables cannot change their state over time. Dynamic Bayesian networks (DBNs) [8] extend BNs by introducing a temporal dimension to represent dynamic systems. DBNs represent the state of the system through “snapshots” or ‘time slices” of the system at each time point, where each “time slice” is a traditional BN.

In a DBN a random variable *X*_*i*_ can assume different values, one for each time point *t*: a “trajectory” is an assignment of values to each variable $X_{i}^{(t)}$ for each time *t*. A number of assumptions are made in order to keep this representation tractable [31].

The first assumption is discretization of time into time slices where system’s measurements are assumed to be collected at regularly spaced time intervals. According to this assumption we can reparametrize the joint probability distribution (using the chain rule) in the following way:

(1) $$ P\left(X^{(0)}, \ldots, X^{(t)}\right) = \prod_{t=1}^{T} P\left(X^{(t+1)}|X^{(0:t)}\right)   $$

From equation (1) it is clear how the distribution over the trajectories is calculated as the product of the conditional distributions of the variables in each time slice given their values in the preceding ones.

The second assumption is the Markovian assumption that the state of *X* at the future time *t*+1 is independent from its past given its present, i.e, for every *t*≥0,

(2) $$ \left(X^{(t+1)} \bot X^{(0:(t-1))} | X^{(t)}\right)  $$

Equation (1) can now be represented compactly as:

(3) $$ P\left(X^{(0)}, \ldots, X^{(t)}\right) = \prod_{t=1}^{T} P\left(X^{(t+1)}|X^{(t)}\right)  $$

We can now formally define a DBN.

#### **Definition2**.

(Dynamic Bayesian network) [31]. A dynamic Bayesian network is a pair (*B*_0_,*B*_→_). *B*_0_ is Bayesian network over a set of random variables *X*_1_…*X*_*n*_ and represents the initial distribution over the states. *B*_→_ is a 2-timeslice Bayesian network (2-TBNs) which provides a transition model from the timeslice t to timeslice t+1. For any desired time span *T*≥0, the distribution over *X*^0:*T*^ is defined as an “unrolled” Bayesian network, where for any *i*=1…*n*:

- the structure and conditional probability distributions of $X_{i}^{(0)}$ are the same for those for *X*_*i*_ in *B*_0_.
- the structure and conditional probability distribution of $X_{i}^{(t)}$ for *t*>0 are the same as those for *Xi*′ in *B*_→_

Is it therefore clear that a DBN represents the state of a system at different time points, but does not implement an explicit representation of time. A DBN for example cannot be queried to obtain a distribution over when a specific event takes place. In a DBN random variables can also be continuous. In this case, we would have a conditional probability function (generally Gaussian) and parameters such as mean and variance associated with each node.

One of the most popular approaches for structural learning of a dynamic Bayesian network is to find the graph structure which maximizes the Bayesian information criterion (BIC) [32], which for a DBN is defined as follows:

(4) $$ \log P\left(\mathcal{D}|\theta\right) - \frac{d}{2} \log N   $$

where *θ* are the estimated parameters of the structure, *d* is the number of parameters and *N* is dimensionality of the data. In equation 4$\log P(\mathcal {D}|\theta)$ describes how well the graph predicts the data while (*d*/2)·*logN* keeps the model’s complexity under control by penalizing the addition of edges to the graph. As can be noted, the BIC does not depend on any *a priori* information. In their general formulation DBNs must respect the acyclicity constraint due to the presence of intra-slice arcs. In order to compare them with CTBNs, learning with DBNs was performed using the REVEAL [33] algorithm, which learns the parent set of each node independently (only forward inter-slice arcs are calculated). Moreover, in this work DBNs are considered in their discrete data version only, as CTBNs cannot handle continuous input data. A survey of the structural learning algorithms for general DBNs can be found in [34].

### Continuous time Bayesian networks

CTBNs cannot be considered a direct extension of DBNs, but a direct comparison naturally arises and helps to better understand the differences between the two approaches. DBNs model dynamic systems without representing time explicitly. They discretize time to represent a dynamic system through several time slices. In [25] the authors pointed out that “*since DBNs slice time into fixed increments, one must always propagate the joint distribution over the variables at the same rate*”. Therefore, if the system consists of processes which evolve at different time granularities and/or the observations are unevenly spaced in time, the inference process may become computationally intractable. CTBNs overcome the limitations of DBNs by explicitly representing temporal dynamics and thus allow us to recover the probability distribution over time when specific events occur. CTBNs have been used to discover intrusion in computers [35], to analyse the reliability of dynamic systems [36], for learning social network dynamics [37], to model cardiogenic heart failure [38] and high frequency trading [39]. However, CTBNs have never been applied to the analysis of molecular data (a preliminary version of this paper appeared in the proceedings of ISBRA 2014 [40]).

A continuous time Bayesian network (CTBN) is a probabilistic graphical model, whose nodes are associated with random variables and whose state evolves in continuous time. The evolution of each variable is conditioned on the state of its parents in the graph associated with the CTBN model. A CTBN consists of two main components: *i)* an initial probability distribution and *ii)* the dynamics which rule the evolution over time of the probability distribution associated with the CTBN.

#### **Definition3**.

(Continuous time Bayesian network). [25]. Let **X** be a set of random variables *X*_1_,*X*_2_,…,*X*_*N*_. Each *X*_*n*_ has a finite domain of values *Val*(*X*_*n*_)={*x*_1_,*x*_2_,…,*x*_*I*(*n*)_}. A continuous time Bayesian network *B* over **X** consists of two components: the first is an initial distribution $P_{\mathbf {X}}^{0}$, specified as a Bayesian network  over **X**. The second is a continuous transition model, specified as:

- a directed (possibly cyclic) graph  whose nodes are *X*_1_,*X*_2_,…,*X*_*N*_; ${Par}_{\mathcal {G}}(X_{n})$, often abbreviated **U**_*n*_, denotes the parent set of *X*_*n*_ in .
- a conditional intensity matrix, $\mathbf {Q}_{X_{n}}^{{Par}_{\mathcal {G}}(X_{n})}$, for each variable *X*_*n*_∈**X**.

Given the random variable *X*_*n*_, the *conditional intensity matrix* (CIM) $\mathbf {Q}_{X_{n}}^{Par(X_{n})}=\mathbf {Q}_{X_{n}|\mathbf {U}_{\mathbf {n}}}$ consists of a set of intensity matrices, one intensity matrix

$${\small\mathbf{Q}_{X_{n}|\textbf{u}}=\left[ \begin{array}{rrcl} -q_{x_{1}|\textbf{u}} & q_{x_{1} x_{2}|\textbf{u}} &. & q_{x_{1} x_{I(n)}|\textbf{u}} \\ q_{x_{2} x_{1}|\textbf{u}} & -q_{x_{2}|\textbf{u}} &. & q_{x_{2} x_{I(n)}|\textbf{u}} \\. &. &. &. \\ q_{x_{I(n)} x_{1}|\textbf{u}} & q_{x_{I(n)} x_{2}|\textbf{u}} &. & -q_{x_{I(n)}|\textbf{u}} \end{array} \right],} $$

for each instantiation **u** of the parents **U**_*n*_ of node *X*_*n*_, where $q_{x_{i}|\textbf {u}}=\sum \limits _{x_{j}\neq x_{i}}q_{x_{i} x_{j}|\textbf {u}}$ is the rate of leaving state *x*_*i*_ for a specific instantiation **u** of **U**_*n*_, while $q_{x_{i} x_{j}|\textbf {u}}$ is the rate of arrival to state *x*_*j*_ from state *x*_*i*_ for a specific instantiation **u** of **U**_*n*_. Matrix $\mathbf {Q}_{X_{n}|\mathbf {U}_{\mathbf {n}}}$ can equivalently be summarized by using two types of parameters, $q_{x_{i}|\textbf {u}}$ which is associated with each state *x*_*i*_ of the variable *X*_*n*_ when its parents are set to **u**, and $\theta _{x_{i} x_{j}|\textbf {u}}=\frac {q_{x_{i} x_{j}|\textbf {u}}}{q_{x_{i}|\textbf {u}}}$ which represents the probability of transitioning from state *x*_*i*_ to state *x*_*j*_, when it is known that the transition occurs at a given instant in time and parents **U**_*n*_ are set to **u**.

Learning the structure of a CTBN from a data set  consists of finding the structure  which maximizes the *Bayesian score* [41]:

(5) $$ \mathbf{score}_{B}\left(\mathcal{G}:\mathcal{D}\right) = \ln P\left(\mathcal{D} | \mathcal{G}\right) + \ln P(\mathcal{G}).   $$

Efficiency of the search algorithm for finding the optimal structure $\mathcal {G}^{*}$ is significantly increased if we assume *structure modularity* and *parameter modularity*. The prior over the network structure $P(\mathcal {G})$ satisfies the structure modularity property if $P(\mathcal {G})=\prod _{n=1}^{N} P\left (Par\left (X_{n}\right)={Par}_{\mathcal {G}}\left (X_{n}\right)\right)$, while the prior over parameters satisfies the parameter modularity property, if for any pair of structures  and ’ such that ${Par}_{\mathcal {G}}(X)={Par}_{\mathcal {G}'}(X)$ we have that $P(\mathbf {q}_{\mathbf {X}}, \theta _{\mathbf {X}}|\mathcal {G})=P(\mathbf {q}_{\mathbf {X}}, \theta _{\mathbf {X}}|\mathcal {G}')$. In [41] the authors combined parameter modularity, parameter independence, local parameter independence and assumed a Dirichlet prior over *θ* parameters and a beta prior over *q* parameters to obtain the following expression of the Bayesian score for a CTBN *B*:

(6) $$ \begin{aligned} \mathbf{score}_{B}\left(\mathcal{G}:\mathcal{D}\right) = \\ \sum_{n=1}^{N} FamScore \left(X_{n}, {Par}_{\mathcal{G}}(X_{n}) : \mathcal{D}\right)  \end{aligned}  $$

where

(7) $$\begin{array}{@{}rcl@{}} \begin{aligned} FamScore \left(X_{n}, {Par}_{\mathcal{G}}(X_{n}) : \mathcal{D}\right) = \\ \ln P\left(Par\left(X_{n}\right)={Par}_{\mathcal{G}}\left(X_{n}\right)\right) + \\ \ln Marg L^{q} \left(X_{n}, \textbf{U}_{n} : {\mathcal{D}}\right) + \\ \ln Marg L^{\theta} \left(X_{n}, \textbf{U}_{n} : {\mathcal{D}}\right). \end{aligned} \end{array} $$

According to [41] $Marg L^{q} (X_{n}, \textbf {U}_{n} : {\mathcal {D}})$ can be written as follows:

(8) $$ \prod_{\textbf{u}} \prod_{x} \frac{\Gamma \left(\alpha_{x|\textbf{u}} + M[x|\textbf{u}] + 1 \right) \tau_{x|\textbf{u}}^{\alpha_{x|\textbf{u}}+1}}{\Gamma \left(\alpha_{x|\textbf{u}} + 1 \right)\left(\tau_{x|\textbf{u}} + T[x|\textbf{u}] \right)^{\alpha_{x|\textbf{u}}+M[x|\textbf{u}]+1}}   $$

while $Marg L^{\theta } (X_{n}, \textbf {U}_{n} : {\mathcal {D}})$ can be written as follows:

(9) $$  \prod_{\textbf{u}} \prod_{x} \frac{\Gamma \left(\alpha_{x|\textbf{u}}\right)}{\Gamma \left(\alpha_{x|\textbf{u}} + M[x|\textbf{u}] \right)}\times \prod_{x' \neq x} \frac{\Gamma \left(\alpha_{xx'|\textbf{u}} + M\left[x,x'|\textbf{u}\right]\right)}{\Gamma \left(\alpha_{xx'|\textbf{u}}\right)}.   $$

where *Γ* is the Gamma function, *M*[*x*,*x*^′^|**u**] represents the count of transitions from state *x* to state *x*^′^ for the node *X*_*n*_ when the state of its parents **U**_**n**_ is set to **u**, while *T*[*x*|**u**] is the time spent in state *x* by the variable *X*_*n*_ when the state of its parents **U**_**n**_ is set to **u**. Furthermore, $M[x| \textbf {u}] = \sum _{x' \neq x} M[x, x' | \textbf {u}]$, *α*_*x*|**u**_ and *τ*_*x*|**u**_ are hyperparameters over the CTBN’s *q* parameters while $\alpha _{xx^{\prime }|\textbf {u}}$ are hyperparameters over the CTBN’s *θ* parameters. However, $Par(\mathcal {G})$ does not grow with the amount of data. Therefore, the significant terms of $FamScore (X_{n}, {Par}_{\mathcal {G}}(X_{n}) : \mathcal {D})$ are $Marg L^{q} (X_{n}, \textbf {U}_{n} : {\mathcal {D}})$ and $Marg L^{\theta } (X_{n}, \textbf {U}_{n} : {\mathcal {D}})$. Thus, given a dataset , the optimal CTBN’s structure is selected by solving the following problem:

(10) $$\begin{array}{@{}rcl@{}} \begin{aligned} \max_{\mathcal{G} \in \textbf{G}} \sum_{n=1}^{N} &\ln Marg L^{q} (X_{n}, \textbf{U}_{n} : {\mathcal{D}}) \\ & + \ln Marg L^{\theta} (X_{n}, \textbf{U}_{n} : {\mathcal{D}}),  \end{aligned} \end{array} $$

where **G**={**U**_*n*_∈**X**:*n*=1,…,*N*} represents all possible choices of parent set **U**_*n*_ for each node *X*_*n*_, *n*=1,…,*N*. Optimization problem (10) is over the space **G** of possible CTBN structures, which is significantly simpler than that of BNs and general DBNs. Indeed, learning optimal BN’s structure is NP-hard even when the maximum number of parents for each node is limited, while the same does not hold true in the context of CTBNs. In fact, in CTBN all edges are across time and represent the effect of the current value of one variable to the next value of other variables. Therefore, no acyclicity constraints arise, and it is possible to optimize the parent set **U**_*n*_ for each variable *X*_*n*_, *n*=1,…,*N*, independently. In [41] the authors proved that if the maximum number of parents is restricted to *k*, then learning the optimal CTBN’s structure is polynomial in the number of nodes *N*. However, we usually do not want to exhaustively enumerate all possible parent sets **U**_*n*_ for each variable *X*_*n*_, *n*=1,…,*N*. In this case we resort to *greedy hill-climbing* search by using operators that add/delete edges to the CTBN structure . It is worthwhile to mention that family scores of different variables do not interact. Therefore, the *greedy hill-climbing* search on CTBNs can be performed separately on each variable *X*_*n*_, thus making the overall search process much more efficient than on BNs and general DBNs.

#### CTBNs for gene network reconstruction

**Figure 1:**
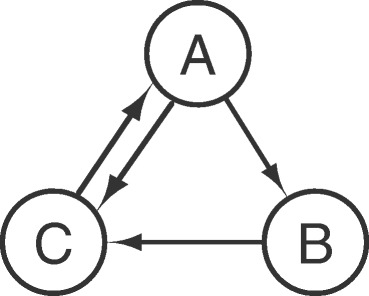
**A simple gene network.**

In a CTBN the amount of time that a gene spends in a given state before switching to a different state plays a central role. This is a key point since the duration of a regulatory interaction is known to be relevant. For example, Th17 cells tend to became pathogenic when the production of Il17a remains protracted for a long time. When cells become pathogenic, the regulatory interactions are different compared to the non-pathogenic phenotype. From this it is clear how the duration of a certain regulatory event can trigger different reactions. The learned structure of a CTBN provides an intuitive and meaningful level of abstraction of the evolution of the regulatory process over time. For instance, a transcription factor which works as permanent hub during the whole process will most likely be at the top of the inferred network hierarchy and is characterized by a high degree of outgoing arcs. On the other hand, transcription factors which act only during some time intervals will likely appear at an intermediate level with both incoming and outgoing connections. Intuitively, genes which are only regulated (i.e. cytokines) will be leaf nodes with only incoming arcs. In the learned network arcs are directed but do not encode information about positive or negative regulation. A direct arc between two genes implies a direct causal relation (regulation) between the pair. Longer paths between two nodes suggest that the influence of one gene on the other pass through a regulatory chain involving intermediate genes. Even if not displayed in the networks, auto regulation interactions, interaction directions (positive/negative) and relative timings are encoded within the conditional intensity matrices (CIMs) associated with each node. Let’s consider the following example consisting of a small network of 3 genes and shown in Figure 1. The three variables are binary; for example the gene *A* can be in either the status *a*_0_ = normally expressed or *a*_1_ = over expressed. The set of CIMs below describes the full dynamic behavior of the system. Specifically, each CIM describes the expected times of transition of a node conditioned to the current state of its parents. Here, we assume the time unit is equal to one minute. If the gene *C* is normally expressed and both its parents *A* and *B* are currently over expressed, then its transient behavior is described by the CIM $\mathbf {Q}_{C|a_{1}, b_{1}}$, which is telling us that the gene *C* is expected to switch from normally expressed to over expressed in 1/0.7 = 1.43 minutes.

$$ \textbf{Q}_{A|c_{0}}=\left[ \begin{array}{cc} -0.1 & 0.1 \\ 0.2 & -0.2 \\ \end{array} \right] \quad \mathbf{Q}_{A|c_{1}}=\left[ \begin{array}{cc} -0.5 & 0.5\\ 0.1 & -0.1 \\ \end{array} \right] $$

$$\textbf{Q}_{B|a_{0}}=\left[ \begin{array}{cc} -0.1 & 0.1 \\ 0.2 & -0.2 \\ \end{array} \right] \quad \mathbf{Q}_{B|a_{1}}=\left[ \begin{array}{cc} -0.5 & 0.5 \\ 0.1 & -0.1 \\ \end{array} \right] $$

$$\textbf{Q}_{C|a_{0}, b_{0}}=\left[ \begin{array}{cc} -0.1 & 0.1 \\ 0.2 & -0.2 \\ \end{array} \right] \quad \mathbf{Q}_{C|a_{0}, b_{1}}=\left[ \begin{array}{cc} -0.5 & 0.5 \\ 0.1 & -0.1 \\ \end{array} \right] $$

$$\textbf{Q}_{C|a_{1}, b_{0}}=\left[ \begin{array}{cc} -0.5 & 0.5 \\ 0.1 & -0.1 \\ \end{array} \right] \quad \mathbf{Q}_{C|a_{1}, b_{1}}=\left[ \begin{array}{cc} -0.7 & 0.7 \\ 0.1 & -0.1 \\ \end{array} \right] $$

From this CIM $\mathbf {Q}_{C|a_{1}, b_{1}}$, the probability distribution over the possible states of *C* can be propagated forward to any continuous point in time, by calculating:

(11) $$ exp\left(\left(\mathbf{Q}_{C|a_{1}, b_{1}}\right) \cdot \Delta t\right)  $$

Where *exp* is the matrix exponential and *Δt* is the difference between the last known state for the parents of *C* and the time *t* for which we want to calculate the probability distribution of *C*. CIMs are learned together with the graph structure and represent the basis for the inference task, which is not directly investigated in this work.

### Granger causality

The Granger causality test was firstly conceived for the economic domain [17] and is based on a linear vector autoregressive model (VAR). The intuitive idea behind it is that an effect never happens before its cause and translated into the GRN domain it can be explained as follows. Suppose we have a sequence of time measurements for the genes *X* and *Y*. *X* is said to Granger cause *Y* if the autoregressive model of *Y* is more accurate when based on the past values of both *X* and *Y* rather than *Y* alone. The accuracy of the prediction is measured through the variance of the prediction error. Let us suppose that we have bivariate linear autoregressive model for the variables *X* and *Y* defined as:

(12) $$   \begin{aligned} X(t) = \sum_{j=1}^{p}{A_{xx,j}X(t-j)} + \sum_{j=1}^{p}{A_{xy,j}Y(t-j)+\epsilon_{x}(t)} \end{aligned}  $$

(13) $$  \begin{aligned} Y(t) = \sum_{j=1}^{p}{A_{yx,j}X(t-j)} + \sum_{j=1}^{p}{A_{yy,j}Y(t-j)+\epsilon_{y}(t)} \end{aligned}  $$

Where *p* indicates the model’s order, e.g. the number of past observations of the time series to incorporate in the autoregressive model. The impact that each one of these observations has on the predicted values of *X* and *Y* is contained in the matrix *A*. *ε* represents the prediction error for the time series (residuals). Considering the first equation, for *Y* to Granger cause *X* the variance of *ε*_*x*_ must be smaller than the variance of *ε*_*x*_ when the *Y* term is removed from the equation. This original GC formulation is meant to uncover causal relationships among two variables; in multivariate systems a pairwise analysis of this kind applied to all possible pairs of variables is limited in the type of causal relationships that can be uncovered. For this reason, this concept was extended [18,42] to the analysis of multivariate data by introducing the concept of conditional GC. Suppose we have a system with three variables, *X*, *Y* and *Z*. Intuitively, the multivariate linear autoregressive model for the variable *X* can be written as:

(14) $$  \begin{aligned} X(t) =&\, \sum_{j=1}^{p}{A_{xx,j}X(t-j)} + \sum_{j=1}^{p}{A_{xy,j}Y(t-j)} \\ &+ \sum_{j=1}^{p}{A_{xz,j}Z(t-j)+\epsilon_{x}(t)} \end{aligned}  $$

In the equation above, *Y* Granger causes *X* if the variance *ε*_*x*_ is smaller than what it would be when the *Y* term is removed from the equation. VAR models have the undeniable advantage of being well-understood and widely applied in many disciplines such as the neurosciences, economics and biology. In this work GC, like in almost the totality of its applications and theoretical investigations, is considered in its formulation which assumes the observations to be taken at regular and fixed time intervals. As underlined in [43], the Granger causality test can be sensitive to the sampling frequency of the time series, with the risk of the results of the test being biased. Many theoretical efforts have been made to extend this formulation to enable it to directly accommodate time. However, most of the contributions remain theoretical and not much investigation has been performed about adequate test statistics [44]. GC is usually applied in its linear version. However, gene expression data is known to contain non-linear features. Many extensions of GC to the non-linear case have been proposed. Hiemstra and Jones [45] investigated a nonparametric test for both linear and non-linear Granger causality in the economic domain (HJ test), resulting in their method being used in a number of subsequent works. However, Diks and Panchenko [46] more recently showed that the HJ test has a tendency to detect spurious non-linear GC. Among other alternatives proposed to deal with nonlinearities are kernel methods [47], with many kernels being proposed and the Gaussian being one of the most common ones. Non-linear extensions of GC have to deal with the issue of overfitting, which makes the statistical interpretation of the results less clear [48]. Moreover, it is known that different nonlinear transformations lead to different results of the GC test [49]. A recent study [50] showed that for Gaussian distributed variables, non-linear GC approaches cannot account for any extra information in the data because a stationary Gaussian autoregressive process is necessarily linear. For these reasons, in this study GC is considered in its linear approximation, which has been found to work well on systems characterized by a large number of variables.

## Results

### Simulated data

Simulated datasets are important for benchmarking the accuracy of gene regulatory network reconstruction as the true network structure is known *a priori*, which is seldom the case with real biological datasets. In this section simulated time course datasets have been used to benchmark the accuracy network reconstruction with GC, DBNs and CTBNs.

The datasets were generated by the same methodology as was used in the DREAM4 competition [51], extracting subnetworks from the known *in vivo* gene networks of *E. coli* [52] and *S. Cerevisiae*. Subnetworks were extracted by randomly choosing a seed node and progressively adding nodes with the greedy neighbor selection procedure, which maximizes the modularity and is able to preserve the functional building blocks of the full network [53].

To ensure robustness, our studies are not based on one single network instance, but are always based on a set of 10 different networks instances. The reconstruction algorithms are tested under several conditions: for increasing number of nodes in the network (network size), for different time points densities in the dataset (time course granularity) and for datasets with time measurements not evenly but unevenly distributed (randomly spaced). The accuracy of network reconstruction was measured using the *F*_1_ measure for binary classification which is defined as:

$$F_{1}=2\cdot\frac{precision \cdot recall}{precision+recall} $$

where $precision=\frac {\text {true positive arcs}}{\text {true positive arcs + false positive arcs}}$ and $recall=\frac {\text {true positive arcs}}{\text {true positive arcs + false negative arcs}}$.

In statistics the *recall* is referred to as *sensitivity* and the *precision* as *positive predicted* value.

#### Benchmarking for increasing network size

**Figure 2:**
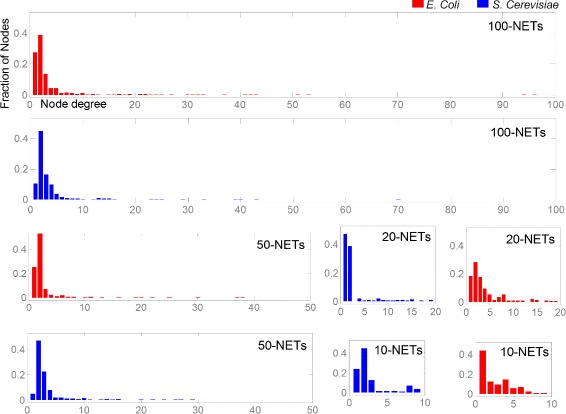
**Degree distribution (in-degree plus out-degree) of nodes in** ***E. coli*** **(red) and** ***S. cerevisiae*** **(blue) for 10-NETs, 20-NETs, 50-NETs and 100-NETs.** Each distribution is obtained from the data of all 10 sampled network instances. X-axis has been shifted up for better visibility. The distribution shows the presence of both large and intermediate hubs indicating that the networks are non-trivial.

The first step of our analysis on simulated data consisted in studying how the three methods perform when faced with the task of reconstructing gene networks of different sizes. From the known *in vivo* gene regulatory network structures of *E. coli* [52] and *S. cerevisiae* we randomly extracted sets of 10 networks consisting of 10, 20, 50 and 100 genes for both organisms. For the sake of brevity, the sets of 10 networks consisting of 10, 20, 50 and 100 genes will be referred to as 10-NETs, 20-NETs, 50-NETs and 100-NETs respectively. Statistical analysis of the complexity of the extracted network structures is provided in Figure 2.

The generated dataset consists of 21 evenly spaced time points. This dataset aims to simulate the amount of data that high-throughput techniques will soon generate while maintaining a realistic time course magnitude: expression microarray experiments repeated with these many time points are today possible. On the other hand, the dataset is still unrealistically rich in terms of number of perturbations and replicates. Such a comprehensive dataset is however necessary to fairly compare the analyzed methods.

Prior to learning, we performed an empirical *optimization* of the model parameters for the three methods; for CTBNs and DBNs this included experimentally establishing the optimum number of discretization levels. More details can be found at the end of this document.

**Figure 3:**
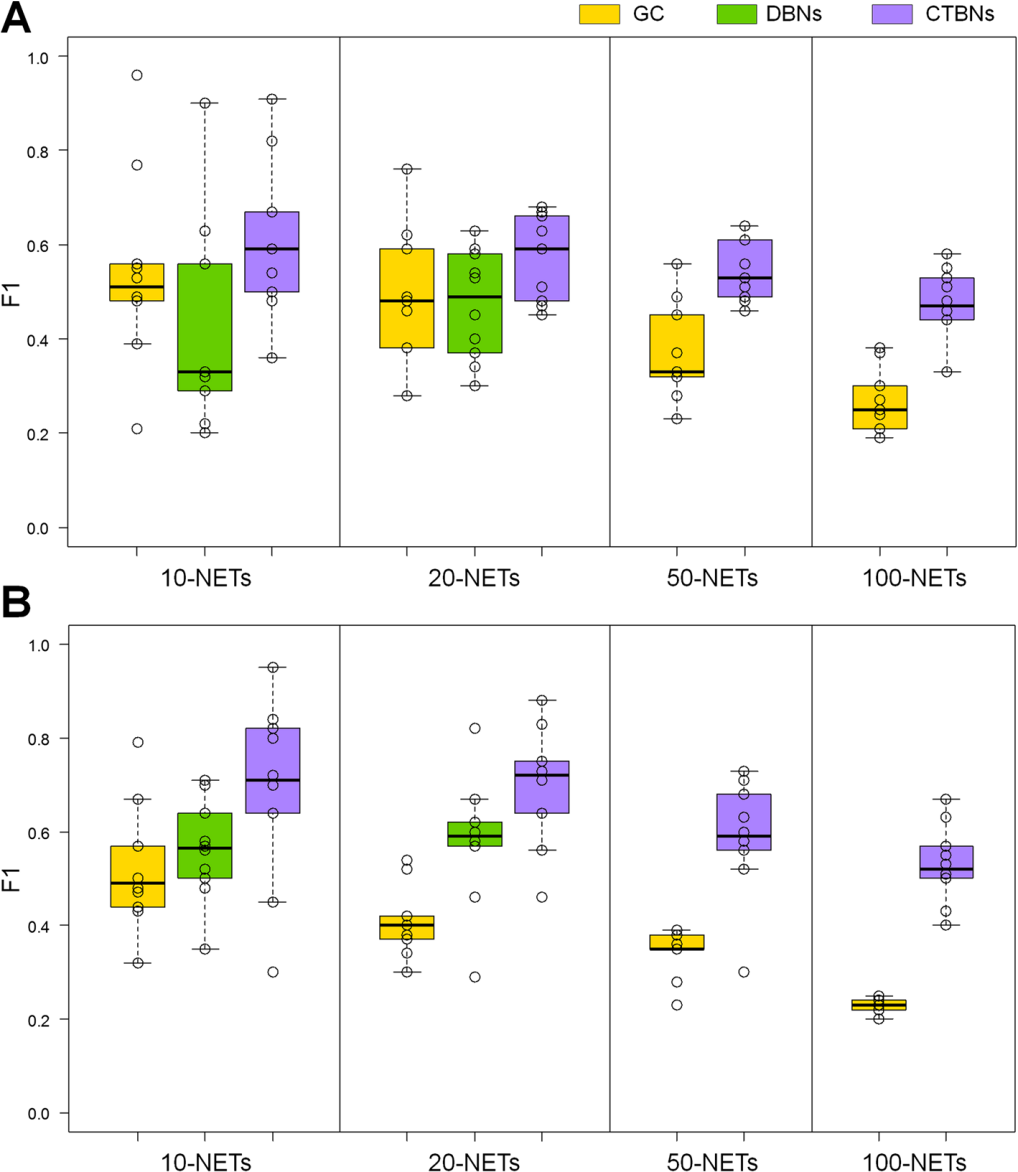
**Performance comparison of CTBNs, DBNs and GC on simulated data for different network sizes.** Organism *E.coli* **(A)** and *S. cerevisiae* **(B)**. Boxplots represents the *F*_1_ values obtained on the 10 sampled network instances of each size, which are also plotted individually as circles.

Results on *E. coli* dataset are summarized in Table 1 (top), where aggregate *F*_1_ values are calculated as the arithmetic mean over the sets of 10 sampled network instances, and Figure 3A, where the individual *F*_1_ values obtained by the methods on the 10 sampled network instances are represented through boxplots. For 50-NETs and 100-NETs learning with DBNs became computationally intractable; therefore, the corresponding results are not available. It can be concluded that the reconstructed network structures were the most accurate for CTBNs which outperformed DBNs and GC for 10-NETs, 20-NETs, 50-NETs and 100-NETs in terms of the mean *F*_1_ values. A paired t-test confirmed that the *F*_1_ values for CTBNs were significantly higher than for DBNs and GC in all tested network sizes (p-value cutoff 0.05). Moreover CTBNs were robust with respect to the increasing network size: their performance smoothly degraded as the number of nodes of the network increased. Indeed, the difference between mean *F*_1_ values for CTBNs and GC increased progressively with the network’s size. GC outperformed DBNs on 10-NETs (0.13 mean *F*_1_ gap) while on 20-NETs GC were only marginally more accurate than DBNs with a limited mean *F*_1_ difference of 0.02.

**Table 1:** **Performance comparison of CTBNs, DBNs and GC on simulated data for different network sizes**

**Method**	**NETs size**	**Mean precision**	**Mean recall**	**Mean** ***F***_***1***_	***F***_***1***_ ** SEM**
GC	10	0.46	0.68	**0.54**	6.40E-02
	20	0.40	0.70	**0.49**	4.33E-02
	50	0.24	0.82	**0.37**	3.23E-02
	100	0.16	0.82	**0.27**	2.13E-02
DBNs	10	0.90	0.29	**0.41**	6.90E-02
	20	0.55	0.42	**0.47**	3.66E-02
CTBNs	10	0.66	0.58	**0.61**	5.13E-02
	20	0.72	0.48	**0.57**	2.79E-02
	50	0.53	0.57	**0.54**	1.95E-02
	100	0.45	0.51	**0.48**	2.28E-02
Random	10	0.16	0.55	**0.24**	2.12E-02
	20	0.11	0.51	**0.18**	1.68E-02
	50	0.03	0.49	**0.06**	4.35E-03
	100	0.02	0.50	**0.04**	1.15E-03
**Method**	**NETs size**	**Mean precision**	**Mean recall**	**Mean** ***F***_***1***_	***F***_***1***_ ** SEM**
GC	10	0.42	0.75	**0.52**	4.18E-02
	20	0.28	0.81	**0.41**	2.32E-02
	50	0.22	0.78	**0.34**	1.58E-02
	100	0.14	0.80	**0.23**	5.24E-03
DBNs	10	0.62	0.53	**0.56**	3.40E-02
	20	0.60	0.57	**0.58**	4.31E-02
CTBNs	10	0.95	0.58	**0.69**	6.08E-02
	20	0.72	0.70	**0.70**	3.86E-02
	50	0.64	0.56	**0.59**	3.84E-02
	100	0.56	0.51	**0.53**	2.65E-02
Random	10	0.18	0.59	**0.27**	2.10E-02
	20	0.07	0.49	**0.12**	1.27E-02
	50	0.05	0.50	**0.08**	4.88E-03
	100	0.02	0.50	**0.05**	2.63E-03

Organism *E.coli* (top) and *S. cerevisiae* (bottom). Aggregate ***F***_***1***_, *precision* and *recall* values are calculated as the arithmetic mean over the sets of 10 sampled network instances, the standard error of the ***F***_***1***_ mean (SEM) is also shown. See also Figure 3.

Results on *S. cerevisiae* dataset shown in Table 1 (bottom) and Figure 3B reaffirmed the same conclusions even more emphatically. CTBNs outperformed DBNs and GC for all network sizes, with the mean *F*_1_ difference between CTBNs and GC increasing from 0.17 for 10-NETs up to 0.29 for 100-NETs. Interestingly, on this dataset DBNs outperformed GC (+0.04 mean *F*_1_ on 10-Nets, +0.17 mean *F*_1_ on 20-NETs). The paired t-test confirmed the significant superiority of CTBNs in all cases over both DBNs and GC. DBNs were significantly better than GC on 20-NETs.

As a negative test we also simulated a *random* reconstruction method which starts with an empty graph and randomly adds edges to it. As expected, this random algorithm had low precision while its recall stabilized around 0.50. As shown in Table 1 the performances of the three methods were always better than the random algorithm, confirming their effectiveness.

#### Benchmarking for increasing time course granularity

The second set of tests are conceived to compare the network reconstruction algorithms with time course datasets of increasing time granularity. Although the overall duration of the simulated experiment was kept fixed, measurements were collected at increasing frequencies (11, 21 and 31) of evenly spaced time points. As in the previous section, datasets were generated for both *E. coli* and *S. cerevisiae*. The network size was kept constant at 20 nodes, as this was seen in the previous section to represent a good trade-off between network complexity and computational cost.

**Figure 4:**
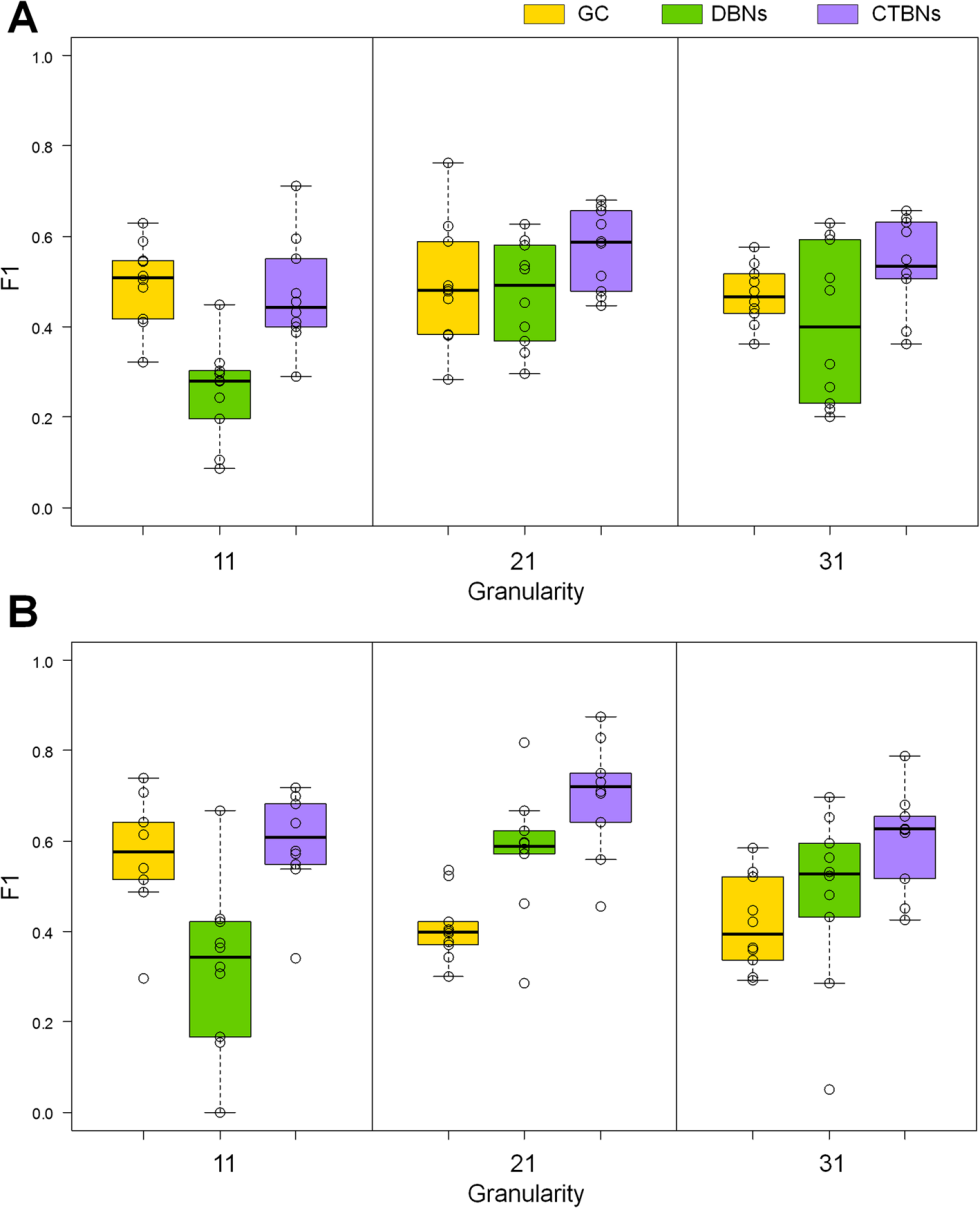
**Performance comparison of CTBNs, DBNs and GC on simulated data for different time granularities on 20-NETs, organism** ***E. coli*** **(A) and** ***S. cerevisiae*** **(B).** The set of 20NETs does not change, what changes is the granularity of the time course data generated from the networks. Boxplots represents the *F*_1_ values obtained on the 10 sampled network instances of each size, which are also plotted individually as circles.

Results on *E. coli* are shown in Table 2 (top) and Figure 4A. Looking at the aggregate *F*_1_ values calculated as the arithmetic average over the sets of 10 network instances (Table 2 (top)) it can be observed that GC appeared to perform consistently, achieving mean *F*_1_ values of 0.50, 0.49 and 0.47 for granularities 11, 21 and 31 respectively, whereas both DBNs and CTBNs achieved their peak performance for a time granularity of 21. DBNs performed poorly (mean *F*_1_ 0.26) for a low time granularity of 11, best for granularity 21 (mean *F*_1_ 0.47) and achieved a slightly lower accuracy for granularity 31 (mean *F*_1_ 0.40). CTBNs achieved a slightly lower accuracy than GC for time granularity 11 (mean *F*_1_ 0.47), achieved the overall best performance for time granularity 21 (mean *F*_1_ 0.57) and had a slightly lower accuracy for granularity 31 (mean *F*_1_ 0.54). A paired t-test over the *F*_1_ values concluded that CTBNs performed significantly better than DBNs for all time course granularities (p-value) and also better than GC (p-value) with the exception of time courses of granularity 11. Finally, GC proved to be significantly better than DBNs for granularity 11, while no statistically significant difference emerged between the two for higher time granularities. The three methods share the trend of reconstruction accuracy initially increasing from time granularity 11 to 21, reaching a peak at 21 and then decreasing for granularity 31: this behavior could be explained by the fact that the *optimal* number of discretization levels has been empirically established for time granularity 21 data and subsequently applied to time granularity 11 and 31 data. The discretization level applied to granularity 31 data may be therefore *suboptimal*.

**Table 2:** **Performance comparison of CTBNs, DBNs and GC on simulated data for different time granularities**

**Method**	**Time course granularity**	**Mean precision**	**Mean recall**	**Mean** ***F***_***1***_	***F***_***1***_ ** SEM**
GC	11	0.43	0.61	**0.50**	2.88E-02
	21	0.40	0.70	**0.49**	3.35E-02
	31	0.35	0.75	**0.47**	3.80E-02
DBNs	11	0.84	0.15	**0.26**	4.33E-02
	21	0.55	0.42	**0.47**	3.66E-02
	31	0.68	0.30	**0.40**	2.79E-02
CTBNs	11	0.70	0.36	**0.47**	2.05E-02
	21	0.72	0.48	**0.57**	5.54E-02
	31	0.59	0.51	**0.54**	3.23E-02
**Method**	**Time course granularity**	**Mean precision**	**Mean recall**	**Mean** ***F***_***1***_	***F***_***1***_ ** SEM**
GC	11	0.47	0.76	**0.57**	4.05E-02
	21	0.28	0.81	**0.41**	5.78E-02
	31	0.29	0.80	**0.42**	3.56E-02
DBNs	11	0.76	0.21	**0.32**	2.32E-02
	21	0.60	0.57	**0.58**	4.31E-02
	31	0.63	0.40	**0.48**	3.86E-02
CTBNs	11	0.60	0.53	**0.60**	3.25E-02
	21	0.72	0.70	**0.70**	6.03E-02
	31	0.56	0.67	**0.60**	3.48E-02

Tests refer to 20NETs, organism *E.coli* (top) and *S. cerevisiae* (bottom). Aggregate ***F***_***1***_, *precision* and *recall* values are calculated as the arithmetic mean over the sets of 10 sampled network instances, the standard error of the ***F***_***1***_ mean (SEM) is also shown. See also Figure 4.

Results on *S. cerevisiae* are shown in Table 2 (bottom) and Figure 4B. GC performed well on time courses of granularity 11, achieving a mean *F*_1_ of 0.57; however, the drop of effectiveness for granularities 21 and 31 was clear with mean *F*_1_ values of 0.41 and 0.42 respectively. CTBNs were always the most accurate achieving mean *F*_1_ values of 0.60, 0.70 and 0.60 for the three time course densities. Again, DBNs performed poorly for granularity 11 (mean *F*_1_ 0.32, with a -0.28 gap from CTBNs), while better for more finely grained data (0.58 and 0.48 mean *F*_1_). With the exception of granularity 11, DBNs outperformed GC, which is the opposite of what we observed for *E. coli* datasets. A paired t-test concluded CTBNs significantly outperformed DBNs for all time granularities and GC for granularities 21 and 31. Interestingly, it is possible to prove the superiority of GC over DBNs for granularity 11, while vice-versa for granularity 21.

It has to be noted that the search for the *optimal* value of the hyperparameters *α* and *τ* has been performed only for the dataset associated with a granularity value equal to 21. These optimal values were subsequently applied to datasets associated with granularity values equal to 11 and 31. While this choice makes the performances achieved by CTBNs *suboptimal*, it also ensures robustness, that is, it implicitly protects from potential overfitting of the hyperparameters.

#### Benchmarking for unevenly spaced time measurements

The third step of our analysis on simulated data consisted in evaluating the performance of the three methods changes when the time measurement are not evenly spaced over time but randomly sampled. This is a typical scenario in wet-lab experiments.

For the purpose of the test, 10 different random time point instances were sampled and used to generate 10 unevenly distributed time course datasets; tests were run on the set of 20-NETs of the organism *E. coli*. We repeated the numerical experiments for time courses of granularity of 11, 21 and 31 (keeping the 10 random time point instances consistent).

**Figure 5:**
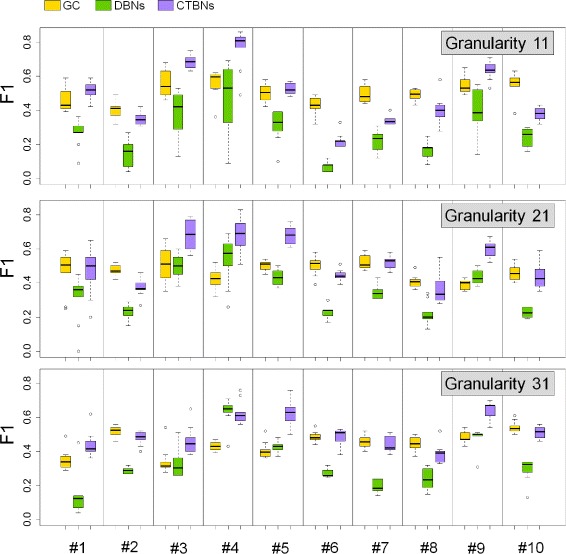
**Performance comparison of CTBNs, DBNs and GC on simulated data for unevenly spaced time points on 20NETs, organism** ***E. coli*** **, for different time course granularities.** Each boxplot represents the *F*_1_ values achieved by the method over the set of 10 unevenly sampled time points instances; the sampled time points are consistent among the three methods. Results are shown separately for each of the 10 network instances of the 20NETs set.

Results are shown in Figure 5 and are consistent for all the three time course granularities (panels A, B, C). For all the network instances, the minimum *F*_1_ value achieved by DBNs among the 10 unevenly (randomly) sampled time point instances is always smaller than the minimum *F*_1_ value achieved by CTBNs on the same 10 unevenly sampled time point instances. Furthermore, the maximum *F*_1_ value achieved by DBNs on the same samples is always smaller than the maximum achieved by CTBNs, for all network instances and time course granularities. The result is clear, showing that CTBNs are always preferable to DBNs when the time course data is not evenly spaced. CTBNs and GC showed comparable ranges of *F*_1_ values (for all granularities), with no clear trend in either of the methods to perform better. GC was better than DBN with respect to both minimum and maximum *F*_1_ values (for all granularities), with only a few cases for which DBNs was preferable.

### Synthetic gene network in *S. cerevisiae*

**Figure 6:**
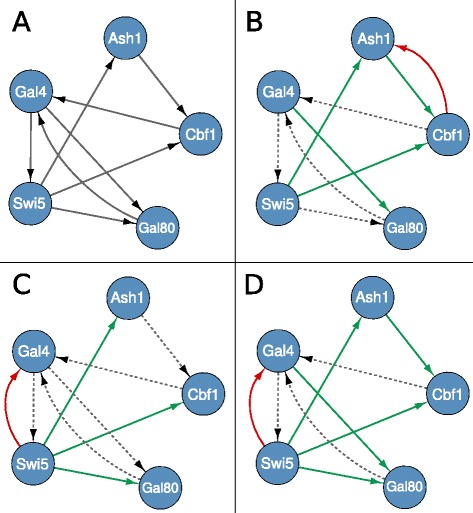
**Performance comparison on** ***S. cerevisiae*** **experimental data.** True network **(A)**, network inferred by GC **(B)**, DBNs **(C)**, CTBNs **(D)**. Green arcs represent true positives, red arcs false positives and dotted lines false negatives.

Due to the current lack of reliable large scale gold standards, *in vivo* evaluation is a critical point for GRN reconstruction methods which often rely on less quantifiable evaluations such as comparison with existing literature and/or information available in public databases. The benchmarking of CTBNs was performed on a small but *certified network*: a network consisting of five genes synthetically constructed in the yeast *S. cerevisiae* [29] and shown in Figure 6 was used. This network, despite its small size, contains a representative set of interconnections including regulator chains and feedback loops. The dynamic behavior of the network was studied by shifting cells from glucose to galactose and vice-versa, and collecting samples every 20 min up to 280 min for the switch-on and every 10 min up to 190 min for the switch-off. 4 and 5 biological replicates were analyzed respectively, gene expression levels were measured through RT-PCR. The authors also made available some interventional data obtained by over expressing each of the five genes in cells grown in either glucose or galactose; however, since only steady-state data was generated for these perturbational experiments, the benchmark was performed on time course unperturbed data alone. On the *S. cerevisiae* experimental dataset the results were coherent with those obtained on simulated datasets: CTBNs outperformed DBNs and GC. A graphical representation of the true network compared with the ones inferred by DBNs, GC and CTBNs is provided in Figure 6. CTBNs achieved both the maximum value of true positives (5) and the minimum value of false negatives (3) while all the three methods made exactly one false positive prediction each.

### Elucidating the regulatory network responsible for murine Th17 differentiation using CTBNs

Gene regulatory networks have been described extensively in the regulation of immune response, but more importantly in the control of inflammation. Inflammation is a multifaceted cellular response critical for the protection of the host against different types of injuries such as infections. However, the dark side of the inflammatory process is represented by tissue damage since inflammatory responses react against self-tissues. Precise regulation of gene expression is extremely important in the context of inflammation for host survival under its own immune activation. In particular, gene regulation of inflammatory cellular differentiation appears essential for fine-tuning of the entire inflammatory response. At the onset of chronic inflammation, Th17 cellular response is of particular interest. Th17 cells produce well-known soluble molecules such as IL17A, IL17F and IL21 which are important for neutrophil recruitment, infection clearance and delivery of antimicrobial peptides. Fine tuning of the Th17 cell differentiation program appears to be pivotal for proper control of over exuberant inflammatory processes in the vertebrate immune system. While some key regulators of the Th17 differentiation are known, a large portion of the regulatory mechanisms controlling this process remains unclear.

Naive T cells (or Th0) can be polarized to differentiate into one of the T helper phenotypes (such as Th1, Th2, or Th17) by exposing them to various polarizing cytokines. The external signals through cytokines drive different regulatory pathways within the cells, and gene regulatory networks involving master regulator transcription genes dictate the final differentiation status. Th0 cells can be programmed to undergo differentiation into the Th17 phenotype by activating transcription factors such as Stat3 and ROR *γ*t through soluble molecules such as IL6, TGF *β*, IL1 *β*. Furthermore, stabilization of the Th17 phenotype requires the activation of IL23R receptor through the innate cytokine IL23 [54].

Here, structure learning of CTBNs is applied to elucidate the gene regulatory network controlling differentiation of murine naive Th0 to the Th17 phenotype. Data for this study is derived from a recently published time course microarray experiment [55] resulting in transcriptional profiles obtained during murine Th17 differentiation. The microarray measurements were taken with Th0 cells cultured in the presence or absence of polarizing cytokines IL6 and TGF *β*1 in two biological replicates. Measurements were taken at 18 time points unevenly spanned over the first 72 hours following induction. Furthermore, separate measurements were taken involving perturbation with the stabilizing innate cytokine IL23 50h from the start of polarization. This dataset is one of the longest and most finely grained time course data ever generated in the T helper differentiation context, with a total of 58 gene expression microarray samples. In order to keep the results interpretable, the analysis was restricted to the representative set of 275 genes individuated by the authors [55] as reflecting as many aspects of the differentiation program as possible. The bioinformatic analysis of raw data and the data discretization process allowed to further narrow down this set to 186 genes (excluding genes whose fold-changes levels were constant among all the time points). More details about the pre-processing steps can be found at the end of this document. Since the goal of this study is to investigate mechanisms which are characteristic of the IL6+TGF *β*1 type and not those regulatory fluctuations which take place independently of the differentiation process (in both Th0 and IL6+TGF *β*1 cells), fold-change values of IL6+TGF *β*1 versus Th0 were used as input data for the learning algorithm.

Two separate networks have been learned: the first one using unperturbed time course series (from fold changes IL6+TGF *β*1 vs. Th0), the second one using the time course series with the addition of the Il23 cytokine into the culture (from fold changes IL6+TGF *β*1+IL23 vs. Th0+IL23). In order to evaluate which mechanisms are relevant to the stabilization of the phenotype, we looked at differences among the two networks. If the perturbations would have been the type of gene knock-outs and/or gene knock downs, the correct way to proceed would have been to learn one single network from both the unperturbed and perturbed data. Here, the perturbation is of a stabilizing nature, e.g. it enhances differentiation process through the activation of additional regulatory mechanisms and the inhibition of others. For simplicity, from now on we will refer to the first network as IL6+TGF *β*1 network and to the second one as IL23 network.

While a few attempts have been recently made to elucidate the molecular mechanisms of the Th17 stabilization following the addition of IL23 [56,57], the validation of the network dynamic is still open to debate. Consequently, the interpretation and validation of results is more difficult on the IL23 network than on IL6+TGF *β*1. For this reason, a large part of the discussion and quantitative validation of the results refers to the IL6+TGF *β*1 network, while only main differences and specific interesting mechanisms that emerged in the IL23 network are discussed.

#### Network validation in absence of gold-standard

CTBNs bring to light the interactions happening in between densely sampled time slices, resulting in a detailed description of all the regulatory steps taking place over the 72 hours differentiation process. Due to the lack of biological analysis with this level of detail, validation through the literature gives evidence that the inferred network is non trivial. Indeed, literature gives evidence that gene interactions are often derived from studies based on static or coarsely grained measurements. As a consequence, what emerges from such studies can be incomplete since the known set of interactions may represent only a subset of all the interactions that are taking place. For this reason, a validation approach that tries to enumerate how many predicted direct interaction are known is not a reliable one. On the other hand, it is known that when considering networks encoding temporal interactions like in the case of CTBNs, the graph can allow cycles. In this situation the presence of an incorrectly inferred arc at some point of the network (something likely to happen) creates a large number of additional paths connecting genes. For this reason, a validation approach which tries to find a pathway between genes known to be related could lead to biased results, where incorrectly inferred arcs paradoxically lead to a greater number of true positives. It is clear that the benchmarking of CTBNs in the absence of a gold-standard cannot be performed in a purely quantitative way, but it has to be complemented with a reasoned biological interpretation of the network.

#### Quantitative validation of the IL6+TGF *β*1 inferred network

**Figure 7:**
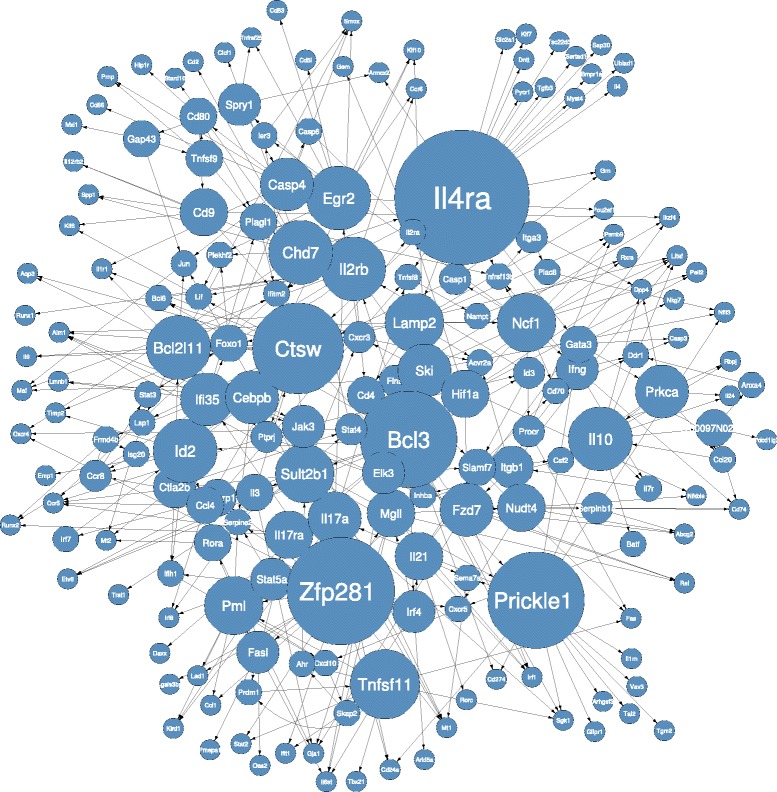
**IL6+TGF** ***β*** **1 inferred network.** Node sizes are proportional to the number of outgoing arcs.

The IL6+TGF *β*1 network inferred from data is shown in Figure 7. The graph is characterized by 186 nodes connected by 365 arcs. For 67 of these arcs solid literature evidence has been found. Only direct known relations were considered, while known relations separated by one or more unknown intermediary nodes were not included in these statistics. A list of these known interactions together with related PubMed IDs is provided in Table 3. Among the listed arcs, 14 appeared in the predicted IL6+TGF *β*1 network with a reverse orientation compared to the literature. This is a well known problem with reconstructing networks referred to as model *non-identifiability*, which arises when given the data, it is not possible to recover (learn) a unique set of parameters. Instead, in such situations we have multiple sets of parameters settings that are indistinguishable given the data [31]. The *non-identifiability* of a model can be due to data scarcity (and/or lack of interventional data) or the presence of hidden variables. Given that we are examining a subset of genes, both hypotheses are possible. For these reasons, the inverted interactions were considered valid.

**Table 3:** **IL6+TGF** ***β*** **1 network validated interactions**

**Source**	**Target**	**PubMed ID**
Il17a	Klrd1	21911461
Il17a	Sgk1	23467085
Il17a	Cd24	19830744
Il21	Rorc	19682929
Stat3	Foxo1	22761423
Irf4	Il21	24430438
Il2rb	Runx1	21292764
Fasl	Rora	19119024
Il10	Ccl20	11244051
Il10	Il7r	18401464
Il10	Rbpj	22933629
Il10	Il24	24130510
Il10	Batf	22992523
Il10	Csf2	24222115
Prkca	Il10	9278292
Stat3	Foxo1	22761423
Foxo1	Smox	22761592
Jun	Maf	22001828
Il4ra	Il4	11918534
Il4ra	Cd30l	11918534
Il4ra	Tgfb3	8601720
Il4ra	Gata3	18792410
Hif1a	Il2ra	23183047
Stat5a	Cxcr5	22318729
Stat5a	Irf8	18342552
Tnfsf11	Prdm1	20133620
Ahr	Tnfsf11	18396263
Egr2	Spry1	21826097
Stat4	Tgfbr1	19808254
Il21	Irf1	19617351
Gata3	Nkg7	19805038
Cebpb	Jak3	12794134
Ifng	Cd74	11009094
Tnfsf8	Nampt	11719441
Csf2	Inhba	12456957
Ccl4	Ccr5	11278962
Bcl3	Irf1	16306601
Bcl3	Id2	22580608
Ncf1	Ifng	15557642
Prdm1	Tnfsf11	20133620
Prkca	Csf2	15661932
Tnfsf11	Fas	12171919
Rora	Mt1h	17666523
Cd80	Cd9	9686645
Elk3	Hif1a	20427288
Foxo1	Timp2	18277385
Bcl3	Il2rb	20235165
Bcl3	Il6st	12969979
Casp1	Tgfbr1	10096572
Ifng	Il7r	18250439
Il2rb	Stat3	9192639
Bcl6	Il2rb	19307668
Ccl20	Il10	20720211
Rora	Stat4	12912921 *
Lamp2	Foxo1	16492665 *
Il2rb	Bcl6	19307668 *
Gap43	Jun	22920255 *
Ctla2b	Stat4	15153495 *
Bcl3	Bcl6	23589612 *
Bcl2l11	Jun	11301023 *
Bcl2l11	Lsp1	23446150 *
Cd9	Spp1	24412090 *
Cxcr5	Cxcl10	22349504 *
Ccl4	Irf8	23853600 *
Ccr8	Stat3	20064451 *
Stat4	Tgfbr1	15879087 *
Sult2b1	Jun	18277385 *

List of direct interactions in the IL6+TGF *β*1 network for which theliterature evidence has been found, together with related PubMed IDs.

An additional assessment of the validity of the inferred network was performed by looking at the leaf nodes (nodes with no children) and the root nodes (nodes with no parents).

In the temporal network semantic leaf nodes are associated with final products (cytokines in our case). In the inferred IL6+TGF *β*1 network 13 of the 90 leaf nodes represented soluble immune mediators, which usually characterize the cells at final steps of their differentiation processes. That was the case of the cytokines *Il4*, *Il9*, *Il24*, *Il1rn*, *Clcf1* and *Tgfb3*, cytokine signal transducer *Il6st* which is shared by many cytokines, cytokine receptors such as *Il12rb2*, *Il1r1*, chemokines such as *Ccl1*, and chemokine receptors such as *Ccr5*, *Ccr6*, *Cxcr4*. Among leaf nodes we also found clusters of differentiations such as *Cd2*, *Cd24*, *Cd274*, *Cd86* which represent a clear marker of the final steps in acquisition of the terminal Th17 phenotype. Furthermore, apoptosis markers like *Casp3*, *Fas*, *Daxx*, *Vav3*, *Trat1*, *Tnfrsf25*, *Tgm2*, *Sertad1* together with programmed cell death 1 ligand 2 (*Pdcd1lg2*) which follow T cell activation and exhaustion were correctly associated with leaf nodes. Transcription factor regulators of late phases of the differentiation processes such as for *Tbet*, *Runx2*, *Runx1*, *Rorc*, *Maf*, all responsible for the final steps of the definition of the Th17 cell phenotype, are correctly placed at the end of the chain. Finally, *Sgk1* is a recently discovered marker identifying the Th17 pathogenic phenotype, acquired by T cell at the late phases of the T cell polarization [58]; in our *Sgk1* network is correctly represented as a leaf node.

Conversely, root nodes are associated with molecules at the beginning of the cascade. Two root nodes were observed at the top of the network structure and both appear to be correctly identified so with their role in initiating the differentiation cascade. One of them is Filamin A (*Flna*), an actin binding and signal mediator scaffolding protein, required for T cell activation in response to TCR activation, also known as “signal1” [59]. The same applies to *Bcl3*, which is known to be activated in response to initial TCR activation [60]. The role of *Bcl3* is discussed more in detail in the next paragraphs, as new interesting insight related to its role emerged from the network.

#### Topological properties and hub nodes of the IL6+TGF *β*1 inferred network

From a topological point of view, the sparsity of the learned causal structure (186 nodes, 365 arcs) is appreciable. From a theoretical point of view, given that the number of variables under study is several order of magnitude greater than the data sample size, network sparsity is something that reconstruction methods seek [61]. A network densely connected may indicate that the learning algorithm is failing to identify true causal relations. Furthermore, sparsity has been shown to be a feature of regulatory networks [26,62]. Even considering that the number of potential arcs was limited by the maximum number of parents allowed per node, which was set to 5, the learned network with 365 interactions (arcs) connecting 186 nodes remains way below this threshold. Another topological feature of the network which emerged is the presence of a few hub nodes regulating a vast number of other genes together and signs of naturally occurring modularity. Both of these features are well-known characteristics of gene networks. Interestingly, modularity has been shown to be a characteristic of static gene networks, but so far modularity has not been studied as a characteristic of networks evolving over time.

**Figure 8:**
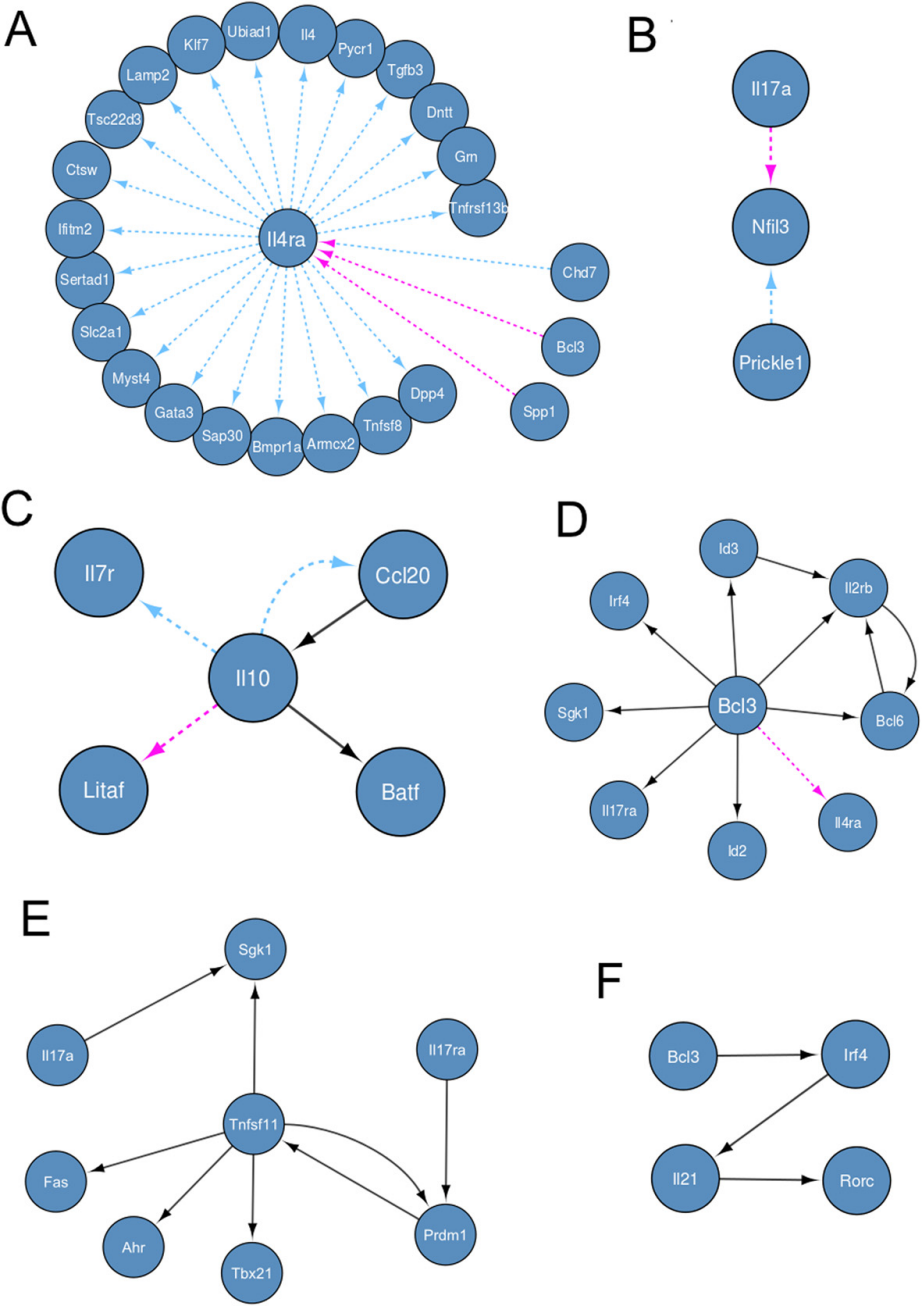
**Some selected interesting known and novel regulatory mechanisms that emerged from the inferred IL6+TGF** ***β*** **1 and IL23 networks.** Panels **A-F** show the selected regulatory interactions which are explained in the discussion section. Light-blue arcs are specific to the IL6+TGF *β*1 network, while pink arcs are specific to the IL23 network. Black arcs are present in both networks.

A major hub node in the network is *Il4ra*, the receptor of the cytokine *Il4*, shown in Figure 8A. Its role in Th2 differentiation is well known, but more interestingly, its preeminent role in regulating Th17 differentiation is a subject of current investigation. Importantly, an inherited polymorphism of *Il4ra* seems to control the ability of the human immune system to regulate the magnitude of Il17 production [63]. Thus, a central role of *Il4ra* in negative regulation of Th17 differentiation is expected [64].

Other major hub nodes include Cathepsin W (*Ctsw*), *Bcl3*, *Zfp281*, *Il4Ra*, *Prickle1* and *Tnfsf11*. Among these *Bcl3* and *Tnfsf11* are known to have a significant influence on Th17 differentiation. *Bcl3*, a member of *IkB* family of proteins, is an essential negative regulator of Toll-like receptor-induced responses and inhibitor of NFkB. Reduced *Bcl3* expression has been associated with Crohn’s disease [65] which is known to be mediated by Th17 chronic expansion. *Bcl3* has an inhibitory role in regulating IL17 release [66]. Indeed, *Bcl3-/-* mice develop autoimmune diabetes with increased Th17 type cytokine expression. Therefore, *Bcl3* is correctly inferred as hub node. *Tnfsf11* alias *Rankl* is known to be a marker of pathogenic Th17 cells in inflammation, and therefore its status as hub in the network is correct [67]. *Ctsw* is a member of the peptidase C1 family, a cysteine lysosomal proteinase that plays a crucial role in the turnover of intracellular proteins as antigens and has a specific function in the mechanism or regulation of CD8 ^+^ T-cell cytolytic activity [68]. However, its role in Th17 differentiation is presently unknown. Similarly, the role of *Zfp281*, a zinc finger transcription factor required in embryonic stems cells for pluripotency [69], and *Prickle1*, a nuclear receptor which is a negative regulator of Wnt/beta-catenin signaling pathway, in Th17 differentiation is yet unknown.

#### Impact of IL23 addition on the differentiation process

**Figure 9:**
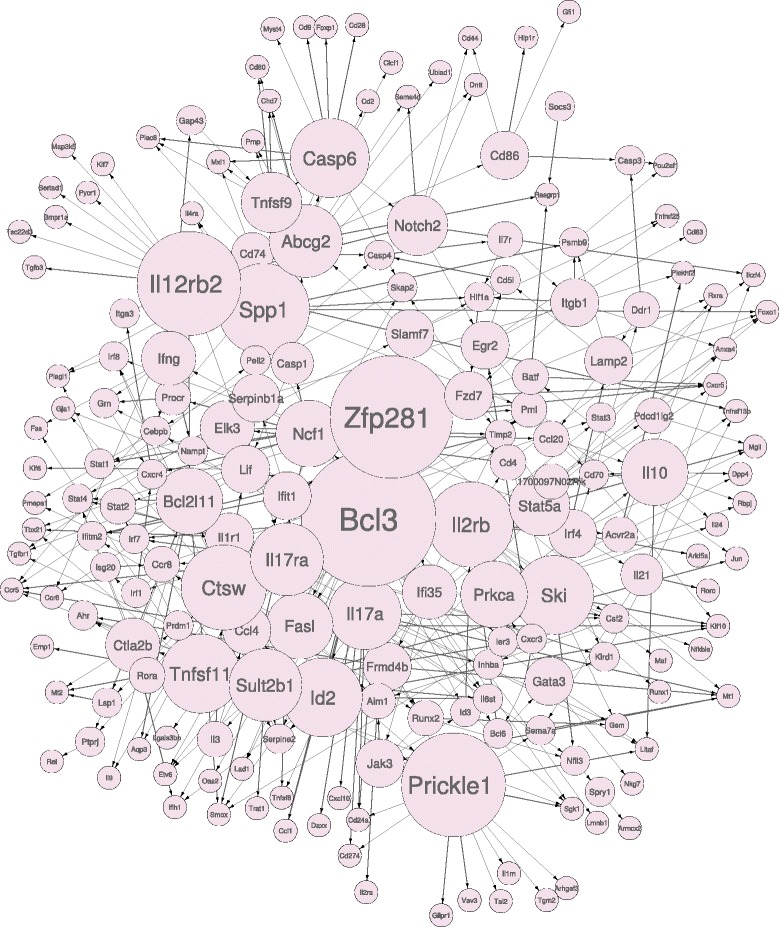
**IL23 inferred network.** Node sizes are proportional to the number of outgoing arcs.

As mentioned, by looking at differences between IL6+TGF *β*1 and IL23 networks we can analyse the impact that the addition of the IL23 cytokine has on the differentiation process. Significant differences emerged between the two networks (IL23 network shown in Figure 9). 165 arcs that were present in the IL6+TGF *β*1 network disappeared in the IL23 network, while 173 new arcs appeared, confirming the widespread impact that IL23 treatment has on the regulatory interactions taking place in the cells [55].

It is interesting to observe how the hub nodes in the IL6+TGF *β*1 network are affected by IL23 perturbation. Considering that the IL23 perturbation represents a positive impulse in Th17 differentiation, it is expected that the IL23 network will not contain hubs that represent a negative regulation of the Th17 differentiation process. This is the case with *Il4ra*, which loses all its outgoing connections and its status as a hub in the IL23 network. On the other hand, IL23 network is expected to have hub nodes which positively regulate the Th17 phenotype. Some newly introduced hubs in the IL23 network include *Il12rb2* and *Il2rb*, both of which are well known for being positive regulators and hubs of the phenomenon [70-72]. *Il2rb* is known to strongly influence the regulation of Th17 differentiation depending on the levels of *Il2* [73]. Another hub node, *Spp1* [74], is particularly interesting because while *Spp1* is known to increase Th17 differentiation, its direct relation with IL23 is still unproven.

Some specific well-known regulatory mechanisms emerged both in the IL6+TGF *β*1 and IL23 networks, together with the new biological insights which can be derived from them, are discussed in the next section.

## Discussion

### Comparative study

For the first time continuous time Bayesian networks (CTBNs) were applied to the gene regulatory network reconstruction task from gene expression time course data. A comparison with two state-of-the-art methods, i.e. dynamic Bayesian networks (DBNs) and Granger causality analysis (GC), was conducted. The performance of the methods was analyzed in three different directions: for networks of increasing size, for time course data of increasing granularity and for evenly versus unevenly spaced time course data.

CTBNs achieved the highest value of the *F*_1_ measure for all network sizes and both *E. coli* and *S. cerevisiae*. Furthermore, they suffered from a limited and smooth loss of performance with respect to the networks of increasing size. This suggests that if applied to networks larger than those analyzed in this paper, CTBNs can still effectively help to uncover the causal structure of the regulatory network. These aspects make CTBNs a good candidate for solving the reconstruction of regulatory networks, which are systems characterized by a large number of variables.

CTBNs were the best performing approach when the time course granularity was sufficiently fine (21 and 31 time points in our experiments), while for coarser granularities (11 time points) CTBNs and GC performed analogously. DBNs performed poorly in the granularity 11 case, showing a big gap from CTBNs and GC on both organisms. The result of CTBNs for granularity 11 was unexpected: probabilistic approaches tend to require a big amount of data in order to be effective.

Thanks to their explicit representation of the time, CTBNs were always preferable to DBNs when the time points were not evenly spaced: the worst case in terms of *F*_1_ value that one can obtain when learning a network from unevenly sampled data (over 10 random samples) is was always better than the worst case obtainable when learning with DBNs. The same favorable situation for CTBNs applied to the best cases. Considerations made for CTBNs over DBNs applies to GC over DBNs as well, while CTBNs and GC showed a similar behavior in response to unevenly spaced data. The poor performance of DBNs on unevenly spaced data is due to the observational model assumption on which their representation of the time is built: variables are assumed to evolve at fixed increments; when that is not the case, time points are treated as evenly spaced with consequent introduction of incorrect information in the model. On the other hand, the good performance of GC on unevenly spaced time course data is surprising; in order to understand the exact reason why GC does not suffer significantly further studies are required. This feature of both CTBNs and GC which emerged is particularly relevant to the gene network reconstruction problem. Indeed, time course data are rarely collected at regular time intervals, while the most common scenario is to have time measurements more densely sampled during some specific phases of the studied phenomenon and coarsely sampled during other phases.

In accordance with what was shown in [28], DBNs and GC were found to perform similarly. In particular, it was not possible to determine if one of these methods was definitively better than the other: for simulated data, GC performed better than DBNs on *E. coli* (Figure 3A) while on *S. cerevisiae* DBNs performed better than GC (Figure 3B). However, when tested on coarsely grained time course data DBNs showed a net loss of performances on both *E. coli* and *S. cerevisiae*, remaining way below the level of accuracy achieved by GC. This result is in contrast with [28] where the authors showed that when the length of the time course is smaller than a given threshold, DBNs outperform GC while vice-versa when the length of the time course is greater than the threshold. However, their test was performed on a 5 genes network, and the authors themselves stated that the results of the test could have changed on networks of larger dimensions.

The simulated time course dataset that we used for the analysis is at present unrealistically rich in terms of the number of perturbations and replicates. However, continuous improvement in experimental technologies will soon allow researchers to reach this level in the near future. When tested on a real experimental dataset of limited dimension and with no interventional data available, CTBNs still achieved the best performance. This result suggest that CTBNs can perform well also on datasets of small dimensions and that they could be suitable for the reconstruction of other types of biological networks as well, such as signaling cascades, where direct manipulation and measurement of the individual members of the cascade are difficult.

### Biological insights emerged from application of CTBNs to Th17 cell differentiation

As follows we discuss some well-known regulatory mechanisms emerged both in the IL6+TGF *β*1 and IL23 networks together with the new biological insights which can be derived from them. For specific direct interactions which are said to be known in the literature, the corresponding reference is omitted in the text but included in Table 3.

#### Negative regulator Il4ra is suppressed upon IL23 addition

As described previously, IL4RA, which mediates a negative role on the Th17 differentiation process, loses its role as a hub node upon IL23 perturbation (Figure 8A). Thus the negative role exerted by *Il4* on Th17 differentiation is suppressed. On the other hand, *Bcl3* and *Spp1* are seen to target *Il4ra* in the IL23 network. Since *Bcl3* and *Spp1* are known to regulate both activation and proliferation of T cells and Th17 differentiation, the interaction between *Bcl3*, *Spp1* and IL23 as suggested by the model is highly plausible.

#### IL23 activates an autocrine loop involving Nfil3

*Nfil3* is a basic leucin zipper transcription factor, known to regulate NK cell differentiation processes and development of NK progenitors [75]. Recently, it has been found that *Nfil3* is required to control the Th17 phenotype by binding the *Rorc* promoter gene and repressing its expression [76]. *Nfil3* is regulated by the circadian clock, which determines the Th17 ability to release Il17a. The interruption of the normal circadian clock reduces *Nfil3* expression leading to a disregulated Th17 with higher *Il17a* expression and occurrence of various inflammatory diseases [76]. The perturbation with IL23 leads interestingly to a change in the *Nfil3* gene interactions: in the IL6+TGF *β*1 network *Nfil3* appear regulated by *Prickle1* (Figure 8B), whose function is still unknown for Th17 differentiation. In the IL23 network, *Nfil3* is regulated by *Il17*. If confirmed, this would further underline the importance of the activation, by IL23 cytokine, of an autocrine loop mediated by Il17. This mechanism is currently unknown and in light of this result may be worth a biological validation.

#### The role of Il10 in Th17 cell differentiation

IL10 is a very well known cytokine, which represents a strong immunoregulator of inflammatory processes. Thus, it is not surprising that in this regulatory network *Il10* represents one of the minor hubs. In particular, the network highlights an interaction/loop already extensively described in the literature between *Ccl20* ligand and *Il10* (Figure 8C). *Il10* is known to be highly expressed in Th17 cells; furthermore the interaction with *Batf* is known as well. A correlation between levels of *Il10* and *Il7r* is also described in T cells. Interestingly, IL23 perturbation here shows that IL23 eliminates this last interaction favoring a new one between *Il10* and lipopolysaccharide-induced TNF-alpha factor (*Litaf*), a DNA-binding protein that mediates the TNF-alpha expression binding to the promoter of the TNF-alpha gene. *Litaf* may be then important to delineate the Th17 pathogenic phenotype, which is achieved thanks to the addition of IL23 in the culture and regulated by *Il10* during Th17 differentiation (Figure 8C). Furthermore, in the IL23 network the loop between *Ccl20* and *Il10* does not appear anymore, which is worth investigating to better understand the function of *Ccl20* in Th17 differentiation.

#### Bcl3 may play a key role in balancing positive and negative markers of Th17 cells

The IL6+TGF *β*1 network shows a central role of *Bcl3*. An interesting and potentially novel interaction between *Bcl3* and *Id3*, a transcription factor involved in T cell development, is suggested (Figure 7D). *Bcl3* is also suggested to interact with *Bcl6* and *Il2rb*. All of these genes are known to be negative regulators of Th17 differentiation [77,78]. In particular, the transcriptional repressor protein *Bcl6* regulates T cell differentiation by repressing Th17 responses and promoting follicular Th cell responses [77]. Interestingly, *Bcl3*, which is also suggested to interacts with *Il4ra* upon IL23 addition, appears to interacts in normal conditions (IL6+TGF *β*1 network) also with *Irf4*, *Sgk1*, *Il17ra* and *Id2*, which are all known as being phenotypic markers of Th17 pathogenic cells [79]. This may indicate a crucial role of *Bcl3* in Th17 differentiation, since it appears to be able to interact and probably affect the balance between positive and negative markers of Th17 cells (Figure 8D). Also, *Bcl3* is revealed by the network as an important regulator of the final Th17 program. *Bcl3* indeed regulates a chain in the network upon IL23 addition (Figure 8F). The interaction between *Il21* and *Rorc* is extensively known, as well as the interaction between *Irf4* and *Il21*. The whole chain seems then to be regulated by *Bcl3*, which as shown before (Figure 8D) is able to regulate other Th17 differetiation markers. Finally, *Rorc* is correctly placed at the end of the chain, as it represents a marker of final differentiated Th17 cells.

#### Prdm1 and Tnfsf11 regulation loop may play a key role in balancing Th17 pathogenic and non pathogenic cells

The IL6+TGF *β*1 network highlights a known interaction between *Tnfsf11* alias *Rankl* and *Prdm1*, alias *Blimp1* (B lymphocyte-induced maturation protein-1) (Figure 8E). *Tnfsf11* is known to be a marker of pathogenic Th17 cells in inflammation whereas *Prdm1* binds to the *Il17a* gene and acts as repressor of *Il17a* expression [80]. The network highlights a loop between *Tnfsf11* and *Prdm1* genes, suggesting an inter-regulation between the two. Interestingly, this interaction is known in other cell types, but not in Th17. The negative feedback loop between the inhibitory transcription factor *Prdm1* and *Tnfsf11* may indicate a balancing mechanism between pathogenic and non pathogenic Th17 cells with *Prdm1* acting as a negative regulator of pathogenic Th17 cells characterized by high expression of *Tnfsf11*. Furthermore, the regulatory chain between *Il17ra*, *Prdm1* and *Tnfsf11* suggests a negative regulation of *Prdm1* on *Tnfsf11* in response to *Il17a*. This is significant considering that *Il4ra* is also hub, which may be an indicator of the importance of cytokine autocrine loops in Th17 differentiation. In other words, this suggests that as in many others systems, Th17 cells autoregulate their differentiation. Finally, according to the prediction, *Tnfsf11* might represent a master regulator of phenotipic markers of Th17 differentiated phenotype since the network underlines its regulation on *Tbx21*, *Ahr*, *Fas*, and *Sgk1*. This last consideration is worth further investigation, since the regulator of finally differentiated pathogenic Th17 cells is not known.

#### Il17a directly regulates Salt-sensing kinase Sgk1

One of the genes which appears to be controlled by *Tnfsf11* is the salt-sensing kinase *Sgk1* (Figure 8E), which has recently been described as a marker of pathogenic Th17 cells [55]. It has been shown recently that environmental factors promote and stabilize Th17 cells and affect their pathogenic role in autoimmune diseases. Sodium chloride has recently been found to drive experimental autoimmune encephalomyelitis (EAE) disease by the induction of pathogenic Th17, thus linking sodium salt intake as an environmental factor influencing the development of autoimmune diseases. In the model proposed in [55], *Sgk1* has been found to be an essential node downstream *Il23* signaling in Th17 differentiation and stabilization. Our network seems to confirm the relevance of *Sgk1* node as it appears to be controlled exclusively and directly by three main hubs (*Bcl3*, *Tnfsf11*, *Prickle1*) and *Il17a* in the IL6+TGF *β*1 as well as in the IL23 network. If experimentally confirmed, this may represent novel information: *Sgk1* would be independent of *Il23* signaling, but dependent on *Il17* itself (Figure 8E). Interestingly, the regulation of *Sgk1* also seems to occur through the receptor of *Il17* (*Il17ra*), through the regulatory chain involving *Prdm1* and *Tnfsf11*. This is aligned with the theory that *Sgk1* depends on *Il17* and may suggest once again the existence of an autocrine loop in the regulation of *Sgk1*.

## Conclusions and future works

The encouraging results achieved in this investigation suggest that structural learning of CTBNs should be considered as a new reliable gene network reconstruction method when time course expression data is available; results indicate that CTBNs would be particularly suitable for the learning of large networks and when the time measurements are not collected at evenly spaced time points. Those are key features which gives a great advantage to CTBNs over the existing state-of-the-art methods. However, CTBNs necessarily require the input data to be discretized. If the data is noisy, as it is usually the case in the biological domain, the discretization can help to reduce the amount of noise. On the other hand, the discretization may also lead to loss of relevant information. Researchers should keep that in mind when using CTBNs.

CTBNs helped elucidate the regulatory network responsible for murine Th17 differentiation, confirming well-known regulatory interactions and main regulators, as well as formulating new biological hyphothesis. Apart from a number of new potential regulators, the network inferred by CTBNs highlighted the presence of several autocrine loops through which Th17 could be autoregulating their own differentiation process. The relevance of this insight comes from the fact that, while self-regulating mechanisms are known to exist in other cell lines, their existence in Th17 has not emerged yet. Wet-lab experiments aimed at validating this hypothesis are now required.

CTBNs assume the duration of the events to be a random variable that is exponentially distributed. The exponential distribution has the characteristics of being *“memoryless”*. CTBNs can be extended to the modeling of systems with memory by introducing hidden nodes/states and representing the system through a mixture of exponential distributions. The application of this extension to the gene network domain is relevant and remains to be explored. Another key aspect to be investigated is the inference task, which would allow for a deeper analysis of the dynamic aspect of the reconstructed gene network, such as answering queries directly involving time. In many biological processes the structure of the causal relationships among variables can vary over time (i.e. there can be different gene networks regulating different phases of the cell cycle). Hererogeneous DBNs [81-83] model the presence of changepoints; that is, specific times when both the structure and the parameters of the network can vary. The area of non-homogeneous processes with CTBNs is yet to be explored.

## Details of numerical experiments

### Simulated data generation

The simulated dataset was generated with the help of the Gene Net Weaver tool [53,84] which has previously been used to generate datasets for network inference challenges of the international Dialogue for Reverse Engineering Assessments and Methods (DREAM) competition [51]. The tool allows the extraction of subnetworks from known *in vivo* gene regulatory network structures of *E. coli* [52] and *S. cerevisiae* [85] endowing them with dynamic models of regulation. When extracting the 10-NETs and 20-NETs, no constraint on the minimum number of regulators (i.e. nodes that have at least one outgoing link in the full network) to include was specified, while for the 50-NETs and 100-NETs this parameter was set to 10 and 20 respectively. This choice on 50-NETs and 100-NETs was made to avoid the generation of networks characterized by a large number of leaf nodes and thus with a too simple structure. No constraint was set on the maximum number of parents allowed per node.

Given each extracted network structure, Gene Net Weaver combines ordinary and stochastic differential equations to generate the corresponding dataset. Perturbations are applied to the first half of the time series and removed from the second part, showing how the system reacts and then goes back to the wild type state. The multiplicative constant of the white noise term in the stochastic differential equations was set to 0.05 as in DREAM4. Finally, all expression values were normalized by dividing them by the maximum mRNA concentration of the related dataset.

### Parameter optimization and data discretization for simulated data

Prior to running the tests on simulated data, an empirical *optimization* of the model parameters for the three methods was run; for CTBNs and DBNs this included experimentally establishing the optimum number of discretization levels. Here all the steps aimed to individuate the best configurations for the three methods here described. It is important to notice that with the term *optimization* we do not refer to the optimization of an objective function, but to a set of independent numerical experiments where the structural learning is run for different values of the model’s parameters. The *optimal* parameters are considered the ones for which the algorithms achieve the highest values of the *F*_1_ measure.

**Figure 10:**
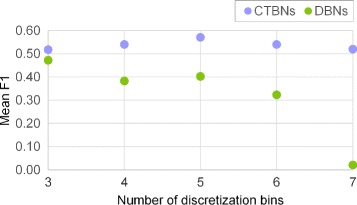
**Impact of different numbers of discretization bins on the performance of CTBNs and DBNs.** Data shown is for organism *E. coli* on 20-NETs.

**Figure 11:**
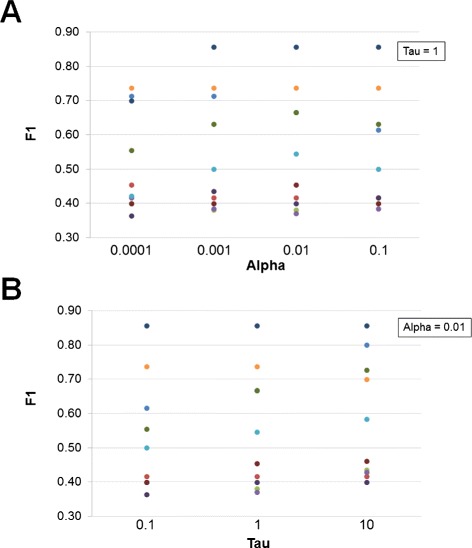
**Sensitivity of network reconstruction performance to hyperparameters** ***α*** **and** ***τ*** **(CTBNs).** Data shown is for organism *E. coli* on 10-NETs. Panel **A** shows the performance variation for different values of *α* when *τ* is kept fixed, while viceversa in Panel **B**. The *F*_1_ value obtained for each of the 10 network instances of the 10-NETs set is represented by a different color.

For CTBNs, *optimization* experiments were run on the 10-NETs and 20-NETs, where the required learning time was still feasible. The optimal parameter values found were subsequently applied to the 50-NETs and 100-NETs. Because CTBNs cannot handle continuous data; a discretization was applied. Discretization of continuous data is known to be a critical task: too few bins (levels) of discretization lead to a loss of important information, while when increasing the number of bins it is known that the required amount of data and computational resources increases as well. To find the optimal number of bins, tests with data discretized into 3, 4, 5, 6 and 7 equal width bins were performed. Best performances were obtained when using 5 equal width bins. It is worthwhile to notice that discretization intervals were chosen individually for each variable (gene) based on the max and min value of expression levels of each variable among the whole set of data generated. In order to preserve the significance and comparability of the results, one needs to keep track of the discretization intervals applied to each variable. The impact of different numbers of discretization bins on the performance of CTBNs and DBNs is shown in Figure 10. An analysis on the importance of the discretization strategy can be found in [5]. Regarding the hyperparameters *α* and *τ*, introduced in section Methods, best values were found to be 0.01 and 5 respectively. Because of the local nature of the learning, the optimal hyperparameters values found on 10-NETs and 20-NETs are expected to be optimal for 50-NETs and 100-NETs as well. Indeed, separate *optimization* process on 10-NETs and 20-NETs returned the same optimal values. Sensitivity of network reconstruction performance to variation of hyperparameters *α* and *τ* (CTBNs) is shown in Figure 11: variations in reconstruction accuracy appeared to be moderate, indicating that performances are robust with respect to values of *α* and *τ*.

The computational nature of the exact structural learning problem lent itself to greedy learning. However, preliminary tests on the 10-NETs returned the same results for both exhaustive and greedy learning, although it cannot be established whether the exhaustive learning on the larger networks would have returned better results. The last parameter investigated was the maximum number of parents allowed for each node: since the greater this value is, the longer is the computational time required, sequential tests with an increasing value of this parameter were run. Interestingly, it was observed that CTBNs were never able to detect more than 3 parents per node even when the true networks contain nodes with a number of parents greater than 3.

For DBNs parameter *optimization* on the number of discretization bins was re-run, and results confirmed that what is optimum for CTBNs may not be the best option for learning with DBNs. Indeed, results indicated 3 as optimum number of discretization bins for DBNs. Discretization intervals were selected individually for each variable as was done for CTBNs. Model selection has been performed by using the BIC criterion [32], which reduces the chance of overfitting. Analogously to what was observed with CTBNs, DBNs were never able to detect more than 3 parents per node. Experiments with 50-NETs and 100-NETs are not shown because the problem became intractable.

For GC analysis no discretization was required since the approach can handle continuous data. Best value for the model order parameter, i.e. the number of past observations to incorporate into the regression model, was discovered to be equal to 1. Covariance stationary (CS) is a requirement for the GC to be applied. Data was CS according to the ADF criterium [86], but not according to KPSS [87]. However when differencing was applied to correct this condition, data interpretation may have become more complicated and in fact performances were significantly worse; as a consequence no differencing has been applied. Pre-processing steps of detrending and demeaning have been applied as well. Analysis was based on the conditional GC test. After performing the GC analysis and obtaining the matrix of magnitudes of GC interactions, the statistically significant set of interactions was selected. The best results were observed with a significance cut-off of 0.01 and a Bonferroni multiple test correction.

Parameter *optimization* was run also with respect to the synthetically reconstructed yeast dataset. Optimal number of bins resulted to be 3 for DBNs and CTBNs, while the maximum number of parents was set to 5. Optimal prior values for CTBNs were equal to those on simulated data. Learning criteria for DBNs was set to BIC. For GC all the pre-processing steps listed for the simulated data were applied, finding a p-value cutoff of 0.05 with an approximation of the False Discovery Rate (FDR) correction being the best performing one.

### Bioinformatic analysis and data pre-processing for murine Th17 data

The microarray raw data for the 275 genes indicated by [55] were analyzed using the Bioconductor package for Affymetrix platform, with annotation chip mouse430a2. Quantile normalization and log2 conversion were performed using RMA. Fold-change values were obtained separately for different biological replicates, assuming the fold-change values being equal to 0 at time point 0. Data was corrected to have mean 0 and standard deviation 1. Supposing *X* to be the fold-change values, noise and random fluctuations in the data resulted to be heavy for *X* < 1.2 and *X* > -1.2; as a consequence, *X* was discretized into 3 different levels: *X*<−1.2, −1.2≤*X*≤1.2, *X*>1.2. Genes whose fold-changes levels after discretization were constant among all the time points were excluded from the analysis.

### Software and tools

Experiments were run using: for CTBNs the CTBN Matlab Toolbox developed at the MAD (Models and Algorithms for Data and text mining) Lab of the University of Milano-Bicocca, for DBNs the Bayesian Net toolbox of Murphy [88] version 1.07, for GC the toolbox for Granger causal connectivity analysis (GCCA) [89] version v2.9.
